# Dietary Intake of 91 Individual Polyphenols and 5-Year Body Weight Change in the EPIC-PANACEA Cohort

**DOI:** 10.3390/antiox11122425

**Published:** 2022-12-08

**Authors:** Mercedes Gil-Lespinard, Jazmín Castañeda, Enrique Almanza-Aguilera, Jesús Humberto Gómez, Anne Tjønneland, Cecilie Kyrø, Kim Overvad, Verena Katzke, Matthias B. Schulze, Giovanna Masala, Claudia Agnoli, Maria Santucci de Magistris, Rosario Tumino, Carlotta Sacerdote, Guri Skeie, Cristina Lasheras, Esther Molina-Montes, José María Huerta, Aurelio Barricarte, Pilar Amiano, Emily Sonestedt, Marisa da Silva, Ingegerd Johansson, Johan Hultdin, Anne M. May, Nita G. Forouhi, Alicia K. Heath, Heinz Freisling, Elisabete Weiderpass, Augustin Scalbert, Raul Zamora-Ros

**Affiliations:** 1 Unit of Nutrition and Cancer, Cancer Epidemiology Research Programme, Catalan Institute of Oncology (ICO), Bellvitge Biomedical Research Institute (IDIBELL), 08908 Barcelona, Spain; 2 Department of Epidemiology, Regional Health Council, IMIB-Arrixaca, 30008 Murcia, Spain; 3 CIBER in Epidemiology and Public Health (CIBERESP), 28029 Madrid, Spain; 4 Danish Cancer Society Research Center, DK-2100 Copenhagen, Denmark; 5 Department of Public Health, Section of Environmental Health, Faculty of Health and Medical Sciences, University of Copenhagen, DK-1353 Copenhagen, Denmark; 6 Department of Public Health, Aarhus University, DK-8000 Aarhus, Denmark; 7 Department of Cancer Epidemiology, German Cancer Research Center (DKFZ), 69120 Heidelberg, Germany; 8 Department of Molecular Epidemiology, German Institute of Human Nutrition Potsdam-Rehbruecke, 14558 Nuthetal, Germany; 9 Institute of Nutritional Science, University of Potsdam, 14469 Potsdam, Germany; 10 Cancer Risk Factors and Life-Style Epidemiology Unit, Institute for Cancer Research, Prevention and Clinical Network—ISPRO, 50139 Florence, Italy; 11 Epidemiology and Prevention Unit, Department of Research, Fondazione IRCCS Istituto Nazionale dei Tumori Via Venezian, 20133 Milan, Italy; 12 Department of Clinical and Experimental Medicine, Federico II University, 80131 Naples, Italy; 13 Hyblean Association for Epidemiological Research (AIRE-ONLUS), 97100 Ragusa, Italy; 14 Unit of Cancer Epidemiology, Città della Salute e della Scienza University-Hospital, 10124 Turin, Italy; 15 Department of Community Medicine, Faculty of Health Sciences, University of Tromsø, The Arctic University of Norway, 9019 Tromsø, Norway; 16 Department of Functional Biology, University of Oviedo, 33007 Oviedo, Spain; 17 Department of Nutrition and Food Science, Campus of Cartuja, University of Granada, 18071 Granada, Spain; 18 Instituto de Investigación Biosanitaria ibs.GRANADA, 18012 Granada, Spain; 19 Institute of Nutrition and Food Technology (INYTA) ‘José Mataix’, Biomedical Research Centre, University of Granada, 18071 Granada, Spain; 20 Navarra Public Health Institute, 31003 Pamplona, Spain; 21 Navarra Institute for Health Research (IdiSNA), 31008 Pamplona, Spain; 22 Ministry of Health of the Basque Government, Sub-Directorate for Public Health and Addictions of Gipuzkoa, 20013 San Sebastian, Spain; 23 Public Health Division of Gipuzkoa, BioDonostia Research Institute, 20014 San Sebastian, Spain; 24 Nutritional Epidemiology, Department of Clinical Sciences Malmö, Lund University, 22184 Malmö, Sweden; 25 Register-Based Epidemiology, Department of Clinical Sciences Lund, Lund University, 22184 Lund, Sweden; 26 Department of Odontology, Umeå University, 90187 Umeå, Sweden; 27 Department of Medical Biosciences, Umeå University, 90187 Umeå, Sweden; 28 Julius Center for Health Sciences and Primary Care, University Medical Center Utrecht, Utrecht University, 3584 CX Utrecht, The Netherlands; 29 MRC Epidemiology Unit, Institute of Metabolic Science, Cambridge Biomedical Campus, University of Cambridge School of Clinical Medicine, Cambridge CB2 0SL, UK; 30 Department of Epidemiology and Biostatistics, School of Public Health, Imperial College London, London W2 1PG, UK; 31 International Agency for Research on Cancer (IARC-WHO), 69008 Lyon, France

**Keywords:** polyphenol, intake, body weight, obesity, cohort, EPIC

## Abstract

Polyphenols are bioactive compounds from plants with antioxidant properties that may have a protective role against body weight gain, with adipose tissue and systemic oxidative stress as potential targets. We aimed to investigate the dietary intake of individual polyphenols and their association with 5-year body weight change in a sub-cohort of the European Prospective Investigation into Cancer and Nutrition (EPIC). This study included 349,165 adult participants from nine European countries. Polyphenol intake was estimated through country-specific validated dietary questionnaires and the Phenol-Explorer database. Body weight was obtained at recruitment and after a mean follow-up time of 5 years. Associations were estimated using multilevel mixed linear regression models. From 91 polyphenols included, the majority (*n* = 67) were inversely associated with 5-year body weight change after FDR-correction (*q* < 0.05). The greatest inverse associations were observed for quercetin 3-O-rhamnoside (change in weight for doubling in intake: −0.071 (95% CI: −0.085; −0.056) kg/5 years). Only 13 polyphenols showed positive associations with body weight gain, mainly from the subclass hydroxycinnamic acids (HCAs) with coffee as the main dietary source, such as 4-caffeoylquinic acid (0.029 (95% CI: 0.021; 0.038) kg/5 years). Individual polyphenols with fruit, tea, cocoa and whole grain cereals as the main dietary sources may contribute to body weight maintenance in adults. Individual HCAs may have different roles in body weight change depending on their dietary source.

## 1. Introduction

Different studies have suggested that polyphenols, bioactive compounds from plants with antioxidant and anti-inflammatory properties, may play a protective role against obesity with adipose tissue and systemic oxidative stress as possible therapeutic targets [1,2]. Excessive production of reactive oxygen species (ROS) leads to oxidative stress which may generate lipid, protein, and DNA damage, inflammation, and alterations in energy homeostasis, as well as promoting adipocyte differentiation and adipogenesis associated with obesity development [2,3]. Approximately 500 individual polyphenols have been identified in the habitual human diet so far, in commonly and regularly consumed plant-based foods, such as fruits, coffee and tea, vegetables, cocoa, or whole grain cereals [4]. Depending on their chemical structure, they can be classified as flavonoids, phenolic acids, stilbenes, lignans, and others [5]. Bioavailability and bio-efficacy differs greatly from one polyphenol to another [6]. Thus, the most abundant polyphenols in our diet are not necessarily those leading to the highest concentrations in the target tissues or the most active ones [6].

Evidence on the link between polyphenols and body weight in humans is not clear, in part due to the heterogeneity between studies (design, populations, and supplements, among others) [7]. Previous observational studies have reported data on polyphenol intake and different anthropometric parameters (i.e., body mass index, waist circumference, and body weight change), mainly studying classes and subclasses. In general, higher intake of polyphenols have been associated with a lesser increase in those parameters. A longitudinal study from the Netherlands reported an inverse association between flavonoid intake and body mass index (BMI) in women [8]. Similarly, a Mediterranean cohort study showed that a higher dietary intake of flavonoids were significantly associated with lower body weight and obesity [9]. The SU.VI.MAX study also reported inverse associations between different flavonoid subclasses (flavanones, flavones), lignans and BMI [10]. They have also shown that those polyphenols, hydroxycinnamic acids (HCAs) and total polyphenols were inversely associated with waist circumference. In a previous investigation, we evaluated associations between the intake of total polyphenols, classes and subclasses, and body weight change in the EPIC-PANACEA cohort [11]. In this previous study, higher intake of flavonoids was inversely associated with body weight change. Conversely, HCAs were the main contributors to total phenolic acids and total polyphenols, all of which were positively associated with body weight gain.

In general, individual polyphenols have been analysed in mechanistic studies that have shown how compounds even from the same class might act differently, or in interventional studies in humans mostly studying different doses of a single compound. For example, catechins, major tea and cocoa polyphenols could be potentially relevant for the prevention of body weight gain and could promote anti-obesity mechanisms, including control of adipocyte differentiation and lipid oxidation and modulation of human gut microbiota [2,12]. Likewise, epigallocatechin from green tea showed effectiveness on body fat and weight reduction in human trials [13]. A review on the relationship between dietary polyphenols from the Mediterranean diet and obesity highlighted the role of several individual compounds, including epigallocatechin gallate, hydroxytyrosol and resveratrol, in molecular mechanisms associated with obesity [7]. However, evidence on other different individual polyphenols and body weight and obesity in humans and from population-based studies is limited.

The current study aimed to delve deeper into the relationships between polyphenols and body weight control, focusing on polyphenols individually.

## 2. Materials and Methods

### 2.1. Population

The European Prospective Investigation into Cancer and Nutrition (EPIC) is a multicentre cohort including ~521,000 men and women from 10 Western European countries with 23 centres, recruited between 1992 and 2000, mainly from the general population. Details of the recruitment and study design have been published previously [14]. The Physical Activity, Nutrition, Alcohol, Cessation of smoking, Eating out of home in relation to Anthropometry (PANACEA) project is a sub-cohort of the EPIC study, where body weight was collected at baseline and at one follow-up (between 2–11 years after the recruitment), making it possible to investigate body weight changes [15]. For the present study we excluded pregnant women, participants with extreme or implausible diet values, with unreliable anthropometric measures, and without lifestyle information at baseline (*n* = 23,713). We also excluded participants with missing body weight values at follow-up (*n* = 121,866) and with extreme/implausible values for annual weight change (≤−5 or >5 kg/year) or body mass index (BMI) (<16 kg/m^2^) at follow-up (*n* = 2066). Finally, data from Greece was not available for the current study (*n* = 24,638). The final analyses included 349,165 participants (Figure S1).

### 2.2. Dietary Data

At baseline, validated country/centre specific dietary questionnaires were used to collect dietary data regarding the previous year [16]. The standardized EPIC Nutrient Database [17] was used to estimate total energy and nutrient intake. Dietary intake of polyphenols was estimated using the Phenol-Explorer database [18], where more than 500 individual polyphenols have been included from over 400 different dietary sources, considering the effects of food processing and cooking. Further details were described previously [19].

### 2.3. Anthropometric Characteristics and Body Weight Change

Two weight measures were available for each participant: one at baseline and one at follow-up. At baseline, anthropometric characteristics were mostly measured by trained specialists using standardized methods [20]. The body weight and height of participants were measured at baseline in most centres, with participants wearing no shoes. The exceptions were the centres from France, Oxford (UK) and Norway, where baseline weight and height were self-reported. At follow-up body weight was mostly self-reported; it was measured only in participants from Doetinchen (the Netherlands) and Cambridge (UK) [20]. Assessment of self-reported weight was conducted through mailed questionnaires, with several exceptions: Spain completed the questionnaire on the phone and Varese used a combination of postal surveys and telephone interviews [21]. The accuracy of self-reported body weight was improved with prediction equations derived from participants from EPIC-Oxford where body weight at baseline was both measured and self-reported [20]. To calculate body weight change, baseline weight was subtracted from follow-up weight, divided by follow-up years to obtain annual weight change and multiplied by 5 to obtain body weight change over 5 years.

### 2.4. Other Covariates

A broad spectrum of sociodemographic, lifestyle, and clinical characteristics were collected through standardized questionnaires [14] at baseline. Physical activity was collected through the validated EPIC-Physical Activity Questionnaire [22] and classified using the Cambridge index [23]. Smoking status was collected at both baseline and follow-up.

### 2.5. Statistical Analysis

Dietary data on 419 individual polyphenols were available for this study. For inclusion in the analysis, a cut-off point was made for polyphenols for which over 50% of the study participants were consumers and with a mean intake ≥ 1 mg/d. A total of 91 individual polyphenols met these criteria and were analysed as continuous variables. To improve right skewness, polyphenol data was log2-transformed, meaning that one unit increase corresponded to a doubling in the intake. To manage very small intakes, values below 0.0001 mg/d were transformed into zero. Then, half the minimum intake of the corresponding polyphenol was added to each zero value. Pearson’s correlation coefficients were calculated between polyphenols and were considered strong when r ≥ 0.8. Correlated polyphenols were clustered in groups. From each group, those individual polyphenols presenting the strongest correlations and the highest median intake were selected (on behalf of the others) for the final analysis. Main food sources were selected from our previous EPIC study, where dietary data at baseline were linked to the Phenol-Explorer database [24]. Distributions of participants’ characteristics were calculated according to quintiles of body weight change over 5 years, with quintiles 2 and 3 as reference categories (i.e., maintenance, range −2.23 to 1.77 kg/5 years). We performed multilevel mixed linear regression models with random effects on the intercept. EPIC centres were taken as random effects to control for differences in follow-up procedures and questionnaire designs among centres. ANOVA and Akaike’s information criterion were used to verify their design. Model assumptions were checked visually by plotting residuals. Missing values were classified as unknown for categorical variables and omitted (*n* = 225) for continuous variables. Restricted cubic splines were used to evaluate linearity of the associations for continuous covariates. Baseline BMI and follow-up years showed a non-linear relationship with body weight change over 5 years. Thus, splines with 3 knots (percentile 10, 50 and 90) were included as covariates for these two variables. Knot positions were determined using the Harrell criteria [25]. We fitted several multivariable-adjusted models that controlled for potential confounders selected a priori, based on previous clinical and epidemiological evidence [26,27,28,29,30]. Model 1 was adjusted for sex, age, and BMI (at baseline). Model 2 was further adjusted for follow-up years, smoking status at follow-up, physical activity, education level, alcohol consumption, and menopausal status. Model 3 was additionally adjusted for energy intake and plausibility of energy intake reporting. The latter included three categories according to the ratio of reported energy intake to predicted basal metabolic rate (EI:BRM): under (EI:BMR < 1.14), plausible (1.14 to 2.1) and over reporters (>2.1), with the use of cut-off points proposed by Goldberg [31]. Finally, we fitted another fourth model adjusted for dietary factors as a proxy of diet quality: vitamin C and fibre intake. An additional analysis for model 4 was performed estimating polyphenols according to energy density. We divided polyphenols by energy intake and multiplied by 2000 (average daily caloric consumption for adults). Interactions were explored between individual polyphenols and different variables that have been shown to influence body weight changes [32,33,34,35]: sex (male vs. female), age (< vs. ≥50 years), BMI categories (underweight, normal weight, overweight, obesity), menopausal status (pre-, post-, and peri-menopausal), smoking status at follow-up (never, former, current), and tertiles of fibre intake, in relation to body weight change. To explore interactions for both fibre and BMI, the categorical variable was added to model 4 (tertiles of fibre intake and BMI categories (underweight, normal weight, overweight, and obesity)). *p* values for interactions were calculated using the likelihood ratio test. Sensitivity analyses were performed excluding participants with chronic diseases at baseline (diabetes, cancer, stroke and/or myocardial infarction, *n* = 57,617), or with self-reported body weight at follow-up (*n* = 320,512). Further analyses were performed with coffee consumers and non-consumers to clarify the role of some polyphenols from coffee in body weight change. For these polyphenols in particular, in secondary analyses, model 4 was further adjusted for sugar, milk, confectionery, and cake intake. Coffee is often consumed together with these foods [36,37,38] which have been shown to increase calorie intake and have therefore been suggested to increase risk of body weight gain when chosen over healthier alternatives [37,39]. The false discovery rate (FDR) was computed to control for multiple comparisons. Differences were considered statistically significant at FDR *q* < 0.05. Statistical analyses were performed with R Statistical Software [40] (version 1.3.1093) using the LmerTest package [41].

## 3. Results

In total, 349,165 participants were included in this investigation, with female participants constituting 73.2% of the sample. Mean age (years) at recruitment was 51.7 (SD = 9.1); mean years of follow-up was 5.1 (2.3) and mean body weight change (kg) over 5 years was 2.6 (5.0). Table 1 shows the distribution of characteristics by quintiles of 5-year body weight change for the total population. The majority of polyphenols were consumed by more than 90% of the population (75 out of 91). The most consumed polyphenols were those from coffee (4-caffeoylquinic acid, median intake: 113 mg/d; 5-feruloylquinic acid: 22 mg/d; and 4-feruloylquinic acid: 16 mg/d), and ferulic acid (22 mg/d), mainly present in whole grain cereals (Table 2 and Table S1). These were followed by hesperidin (16 mg/d) mainly present in citrus fruits and (+)-catechin (11 mg/d), mostly present in cocoa and tea. The highest level of non-consumers was observed for sanguiin H-6, a polyphenol widely present in raspberries (non-consumers = 48.6%), followed by some polyphenols present in black tea, such as theaflavin, or kaempferol and quercetin 3-O-glucosyl-rhamnosyl-glucoside (non-consumers = 40.4%) (Table 2 and Table S1).

**Figure 1:**
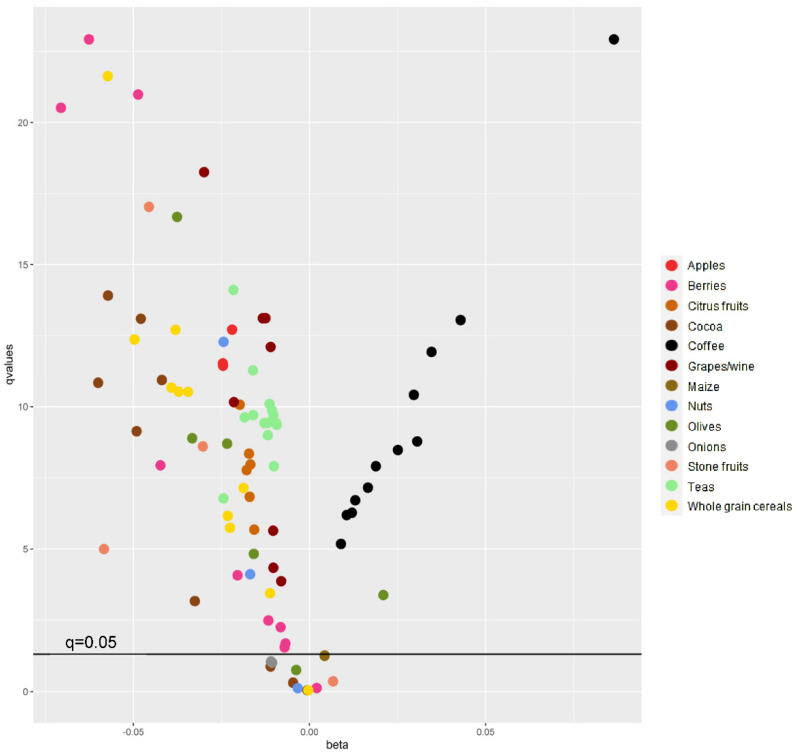
False discovery rate (FDR) *q* values and beta values (kg/5 year) of associations between 91 polyphenols and 5-year body weight change and their main food sources. Beta values correspond to a doubling in intake. *q* values (adjusted *p* value for FDR) are shown as -log10-transformed values to improve visualization. ^1^ Multilevel linear mixed model with random effects on the intercept according to EPIC centre adjusted for age, sex, body mass index (3-knot restricted cubic spline), follow-up time in years (3-knot restricted cubic spline), alcohol intake (g/d), education level, physical activity level, smoking status at follow-up, menopausal status, total energy intake (kcal/day), plausibility of dietary energy reporting, vitamin C intake (mg/d), and fibre intake (g/d) (Model 4).

**Figure 2:**
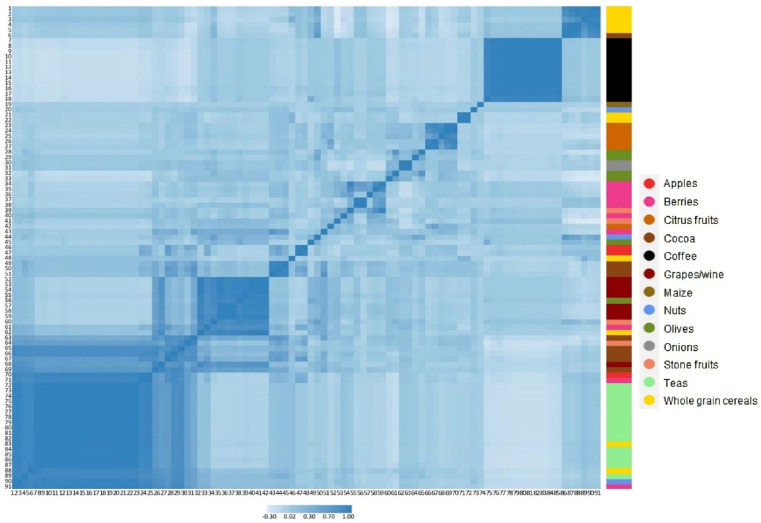
Pearson’s correlation heat-map between the intakes of 91 polyphenols in the EPIC-PANACEA population. Numbers (1 to 91) represent individual polyphenols (Table S12). The right panel shows their main food sources.

Figure 1 shows *q* values (FDR) and beta coefficients for doubling in intake of the 91 polyphenols for model 4, with their respective main food source. Most polyphenols (*n* = 80) showed statistically significant associations with body weight change, of which 67 were inversely and 13 were positively associated with body weight change. Figure 2 shows polyphenol–polyphenol correlations for the 91 individual compounds, as well as their main food sources. After performing Pearson’s correlations, 35 polyphenols with coefficients ≥ 0.8 were separated into six correlation groups. From each group, polyphenols with the highest median intakes were chosen. Thus, six individual polyphenols were selected on behalf of the 35, and therefore 29 were excluded (Table S1). A total of 62 polyphenols remained after excluding highly correlated polyphenols, of which 51 were significantly associated with body weight change and 11 showed null results after adjusting for potential confounders and correcting for multiple comparisons (Table 2 and Table S2). From these, 46 polyphenols were inversely associated with 5-year body weight change for a doubling in intake. Their main dietary sources were fruits, tea, cocoa and whole grain cereals. The greatest inverse associations were observed for a doubling in intake of quercetin 3-O-rhamnoside: −0.071 kg/5 year (95% CI: −0.085; −0.056), quercetin 3-O-glucoside: −0.062 (−0.074; −0.051) and (+)-catechin: −0.060 (−0.077; −0.043) (Figure S2).

Only 5 out of 51 individual polyphenols showed positive associations with body weight gain, in general with coffee as their main food source, except for sinapic acid, for which the main food source was olives (Table 2 and Table S2). Classified as a hydroxycinnamic acid (HCA), doubling in intake of 4-caffeoylquinic acid showed the greatest positive associations with body weight gain for model 4: 0.029 kg/5 year (95% CI: 0.021; 0.038). Results remained the same after further adjusting these five polyphenols for sugar, milk, confectionery, and cake intake. We performed extra analysis including only coffee non-consumers (*n* = 25,414), for which main dietary sources of these polyphenols were plums, berries and black tea. Median intakes of HCAs and 4-caffeoylquinic acid from other dietary sources than coffee, in coffee non-consumers, were 124 and 3 mg/d, respectively. Median intakes in coffee consumers were 520 mg/d for HCAs and 120 mg/d for 4-caffeoylquinic acid. Associations were not statistically significant for coffee non-consumers. In coffee consumers (*n* = 323,751), we observed positive associations with body weight gain for the five polyphenols (Table S3).

Our findings were robust and remained statistically significant for the majority of polyphenols after the sensitivity analyses, excluding participants with chronic diseases at baseline or with self-reported body weight at follow-up (Table S4). After estimating polyphenols according to energy density, results were consistent and very similar to those obtained in model 4 (Table S5). Only statistically significant interactions are reported in Tables S6–S11. According to sex, 29 polyphenols showed significant interactions: the majority (*n* = 19) showed larger negative beta values for men versus women (Table S6). For age as a categorical variable (<50 vs. ≥50 years), significant interactions were observed for 27 polyphenols. The majority (*n* = 16) showed lower beta values for younger participants (Table S7). According to BMI categories, 35 polyphenols showed statistically significant interactions.

The lowest beta values were observed for the categories normal and overweight, versus underweight or obesity (Table S8). Predominantly post-menopausal women had the biggest negative beta values when interactions were explored by menopausal status (Table S9). According to the category of smoking status at follow-up, the most noteworthy were those polyphenols classified as HCAs (4-caffeoylquinic acid, 3-feruloylquinic acid, 4-feruloylquinic acid, 5-feruloylquinic acid) which showed positive associations with body weight gain for non-smoking participants (Table S10). With regard to tertiles of fibre consumption, 30 polyphenols resulted in significant interactions. In general, higher intakes (tertiles 2 and 3) showed lower beta values than tertile 1 (Table S11). All interactions were also penalized for multiple testing using FDR.

## 4. Discussion

In this large prospective cohort, we found that of 51 individual polyphenols investigated, doubling in intake of 46 of these polyphenols was inversely associated with 5-year body weight change. Consumption of quercetin 3-O-rhamnoside, quercetin 3-O-glucoside, both included in the flavonol subclass, and (+)-catechin and procyanidin dimer B3, included in the flavanol subclass (all of them classified as flavonoids), showed the strongest inverse associations. Conversely, the intake of five polyphenols from the HCAs subclass (class phenolic acids) was positively associated with 5-year body weight gain. Investigating polyphenols individually enables the identification of how relatively similar compounds that belong to the same class, may behave differently in relation to body weight change. Unlike classes and subclasses, this approach can highlight some minor individual compounds that may not be the most abundant ones in the diet but may have a relevant association with body weight changes.

A pooled analysis of randomised-controlled trials (RCTs) showed that the current evidence on the effect of quercetin on body weight is inconclusive, and its effects on body weight have not yet been assessed as primary outcome for a large number of intervention studies [42]. As we observed that higher intakes of quercetin glycosides were inversely associated with body weight gain, it may be an interesting compound to study in future clinical trials on body weight. Our results also showed that (+)-catechin was correlated with (-)-epigallocatechin 3-O-gallate (EGCG), a flavanol widely present in tea. A meta-analysis of RCTs analysed the effects of green tea catechins supplementation on body weight [43] and found a positive effect on body weight loss and maintenance (average effect size = −1.31 kg; *p* < 0.001). A systematic review of RCTs showed that daily intakes of green tea, and therefore high doses of EGCG, presented beneficial results in 12-week weight loss interventions [13]. Likewise, an RCT showed that eight weeks of 44 mg daily of green tea catechins (~1 cup of tea) reduced body weight and increased energy expenditure and fat oxidation in participants with obesity [44]. A meta-analysis of RCTs concluded that supplementation with grape seed extract, mainly consisting of catechins, epigallocatechins, and procyanidin dimers, demonstrated a significant improvement in obesity-related cardiometabolic biomarkers [45]. Grape seed extract is also rich in other polyphenols such as resveratrol. Even though we did not include it due to the intake cut-off point, several RCTs demonstrated that resveratrol intake reduces body weight, BMI, waist circumference and body fat [46]. It has been reported that dietary polyphenols have potential for acting on mitochondrial dysfunction and inflammation, as well as kidnapping free radicals, increasing the activity and expression of antioxidant enzymes and inhibiting ROS-producing ones [2]. In addition, polyphenols control adipocyte differentiation and lipid metabolism and oxidation through decrease in the activity of the pancreatic lipase and permeability of the intestine, and through their interaction with the gut microbiota [2]. Recent evidence suggests that gut microbiota-derived polyphenol metabolites may affect appetite control and body weight management [47], and can modulate the development of adipose tissue and the obesity-induced inflammatory genes [48]. However, as far as we know, several of the individual compounds included in this investigation are not yet studied neither by observational nor by interventional studies in humans assessing body weight changes, or evidence on their influence on body weight is still scarce.

Out of 51 individual polyphenols, five compounds classified as HCAs were positively associated with body weight gain: 4-caffeoylquinic acid, 3-feruloylquinic acid, 4-feruloylquinic acid, 5-feruloylquinic acid, and sinapic acid. Most of them have coffee as the main dietary source, except for sinapic acid (mainly from olives). For coffee non-consumers, there was little evidence that these polyphenols were associated with body weight change. As coffee is often part of the Western diet, a pattern rich in sugar and saturated fat [38] that has been associated with body weight gain and obesity [49], we performed an additional analysis adjusting for sugar, milk, confectionery, and cake intake. However, beta coefficients remained the same after this adjustment. In our previous study, we also analysed caffeinated and decaffeinated coffee as exposures, as caffeine is a dietary component that might be associated with body weight loss [50]. We observed higher values for decaffeinated (body weight gain for doubling in intake: 0.012 kg/5 year; 95% CI: 0.007, 0.016) versus caffeinated coffee (0.005; 95% CI: 0.002, 0.009) [11]. Beyond this, mechanistic evidence for these positive associations is lacking and further research is needed.

Despite having found significant interactions with different confounding variables, these results must be interpreted with caution. Differences between subgroups might be due to the large number of participants included in this study. Thus, a minimum change in beta values shows statistically significant values, even though, in general, categories of the same variable followed the same trend. Interactions have been discussed in more detail in our previous study regarding polyphenol classes and subclasses and body weight change in the EPIC-PANACEA cohort [11].

The prospective design of this investigation, its large sample of participants from different European countries, and its sufficient statistical power is a major strength. However, this study has some limitations. Assessing the relationship between diet and health outcomes is challenging in epidemiological studies, due to biases linked to dietary measurement errors [51]. To minimize them, we included validated dietary questionnaires and a standardized database to assess food and nutrient intakes [16,17], as well as the use of Phenol-Explorer database [18] to estimate polyphenol intakes. We had data on a large variety of variables related to diet and lifestyle, but most of them were only collected at baseline, and therefore changes during the follow-up were not accounted for. However, when possible, we took variables measured at follow-up, such as smoking status. As in every observational epidemiological study, residual confounding must be considered. Another limitation is that most of the centres used self-reported body weight at follow-up, a value which usually tends to be underestimated [52]. Average weight gain was higher in EPIC centres where follow-up weight was measured. Thus, Oxford-corrected body weight was used to improve the accuracy of these values. Moreover, after performing a sensitivity analysis excluding participants who self-reported body weight at follow-up, results followed the same trend as in the total population for the majority of polyphenols. The large sample size of this study is both a strength and a limitation, as beta coefficients obtained should be interpreted with prudence: statistically significance is easy to obtain with such a large sample, even after FDR-correction. Still, a daily serving of polyphenol-rich foods can often provide a variety of individual polyphenols and higher quantities than the daily medians assessed in this investigation (e.g., 200 g of apple can provide ~3 mg of quercetin-3-O-rhamnoside [17], whereas its median intake in our population was ~1 mg/d).

## 5. Conclusions

Our results suggest that choosing polyphenol-rich foods, such as fruits, tea and whole grain cereals, may contribute to 5-year body weight maintenance (or less gain) in European populations. These results also suggest that individual HCAs may have different roles in body weight change depending on their dietary source. Our findings provide information about which individual polyphenols may be relevant for future mechanistic studies and interventional studies on body weight change.

## Figures and Tables

**Table 1:** Baseline characteristics of the study population by quintiles of body weight change after 5 years.

Characteristics	Total Population	Q1:	Q2 + Q3:	Q4:	Q5:
		Loss	Maintenance	Moderate Gain	High Gain
Participants (n)	349,165	69,834	139,665	69,842	69,824
Body weight change (kg/5 years) (range)	−24.99; 24.93	−24.99; −2.22	−2.23; 1.77	1.78; 4.16	4.17; 24.93
Body weight change (kg/5 years)	2.62 (5.03)	−6.01 (3.96)	−0.005 (1.09)	2.89 (0.68)	7.56 (3.45)
Age (years)	51.7 (9.1)	53.1 (9.0)	52.1 (9.0)	51.1 (9.1)	50.1 (9.3)
Weight (kg)	69.4 (13.4)	75.2 (14.3)	67.6 (12.7)	67.1 (12.4)	69.4 (13.1)
BMI (kg/m^2^)	25.1 (4.1)	27.2 (4.5)	24.5 (3.8)	24.3 (3.7)	25.0 (4.0)
Follow-up time (years)	5.1 (2.3)	4.4 (1.9)	5.5 (2.4)	5.4 (2.4)	4.8 (2.1)
Alcohol intake (g/d), median (IQR)	11.8 (16.7)	13.6 (19.3)	11.9 (16.1)	11.2 (15.7)	10.7 (16.1)
Energy intake (kcal/d), median (IQR)	2076.2 (606.1)	2073.2 (615.4)	2086.1 (596.7)	2087.2 (602.3)	2048.6 (618.0)
Vitamin C intake (mg/d), median (IQR)	111.7 (73.6)	111.4 (75.5)	111.5 (72.8)	112.0 (72.0)	112.0 (75.9)
Fibre intake (g/d), median (IQR)	22.0 (9.6)	22.0 (10.0)	22.2 (9.5)	21.9 (9.4)	21.7 (9.5)
Sex (female) (%)	73.2	67.2	73.6	75.2	76.5
Education level (%)					
None	4.2	7.9	3.2	2.8	3.6
Primary school	23.2	27.1	23.2	21.5	21.0
Technology school	22.2	22.9	22.2	21.5	22.2
Secondary school	21.7	15.7	21.6	24.7	25.1
Longer education	25.1	22.8	26.3	26.0	24.2
Unknown	3.6	3.6	3.5	3.5	3.9
Physical activity level (%)					
Inactive	18.6	22.6	17.5	17.2	18.4
Moderately inactive	34.2	33.9	34.4	34.8	33.5
Moderately active	27.4	24.1	28.0	29.6	28.5
Active	18.2	18.3	18.6	17.8	17.5
Unknown	1.6	1.1	1.5	1.6	2.1
Smoking at follow-up (%)					
Never	48.3	46.9	48.7	50.1	47.3
Former	30.2	28.6	29.0	30.0	34.2
Current	17.3	20.9	16.8	15.8	16.3
Unknown	4.2	3.6	5.5	4.1	2.2
Prevalent disease ^1^ (%)					
No	83.5	82.7	84.4	84.3	81.8
Yes	7.6	10.0	7.1	7.0	7.9
Unknown	8.9	8.3	8.5	8.7	10.3
Menopausal status ^2^ (%)					
Pre-menopausal	32.6	29.7	31.3	34.0	36.4
Post-menopausal	43.7	49.3	45.7	41.3	37.2
Peri-menopausal	20.6	17.2	19.9	21.8	23.6
Surgical post-menopausal	3.1	3.8	3.1	2.9	2.8

Data are expressed as mean (SD) and collected at recruitment if not stated otherwise. BMI: body mass index; IQR: interquartile range; Q: quintile. ^1^ Diabetes, cancer, stroke and/or myocardial infarction at baseline. ^2^ Only for female participants (*n* = 255,730).

**Table 2:** Change in body weight over 5 years according to baseline polyphenol intake in 349,165 participants from the EPIC-PANACEA cohort.

Individual Polyphenols (*n* = 62) ^1^	Intake (mg/d),	N-C (%)	Model 4 beta (95% CI) ^2^	q-Value ^3^	Main Food Sources ^4^
	Median (p5; p95)				
(-)-Epicatechin 3-O-gallate	3.3 (0.0; 66.2)	0.2	−0.018 (−0.023; −0.013)	<0.001	Coffee, tea, SF
(+)-Catechin	10.9 (2.2; 35.7)	0.0	−0.060 (−0.076; −0.043)	<0.001	Apples, pears, chocolate
2,5-di-S-Glutathionyl caftaric acid	0.3 (0.0; 6.5)	23.4	−0.012 (−0.015; −0.009)	<0.001	Whole grain cereals, rice
3,4-DHPEA-EDA	0.4 (0.0; 4.1)	1.0	−0.022 (−0.031; −0.016)	<0.001	Tea, wine, FV
3-Feruloylquinic acid	8.0 (0.2; 22.7)	0.0	0.026 (0.018; 0.033)	<0.001	Coffee, SF, berries
3-p-Coumaroylquinic acid	1.0 (0.1; 11.4)	0.4	−0.030 (−0.040; −0.021)	<0.001	Whole grain cereals, rice
4-Caffeoylquinic acid	113.2 (2.9; 328.4)	0.0	0.029 (0.021; 0.038)	<0.001	Coffee, tea, legumes
4-Feruloylquinic acid	16.0 (0.1; 46.1)	0.1	0.017 (0.011; 0.023)	<0.001	Coffee, tea
4-Hydroxybenzoic acid	0.4 (0.0; 4.0)	0.0	−0.033 (−0.044; −0.023)	<0.001	Wine, grapes
4-p-Coumaroylquinic acid	1.3 (0.0; 8.6)	2.6	−0.022 (−0.027; −0.015)	<0.001	Tea, apples, pears
5-Feruloylquinic acid	21.8 (0.2; 63.0)	0.1	0.019 (0.012; 0.025)	<0.001	Whole grain cereals, rice
5-Heneicosenylresorcinol	0.23 (0.0; 6.5)	19.9	−0.000 (−0.005; 0.004)	0.873	Wine, cocktails, sauces
5-Heneicosylresorcinol	9.5 (0.4; 28.8)	0.1	−0.039 (−0.050; −0.028)	<0.001	Apples, pears, FJ
5-Heptadecylresorcinol	2.1 (0.2; 17.1)	0.8	−0.038 (−0.048; −0.029)	<0.001	Soups, cakes, biscuits
5-Nonadecylresorcinol	8.5 (0.3; 30.4)	0.1	−0.037 (−0.048; −0.027)	<0.001	Coffee, cream desserts
5-O-Galloylquinic acid	1.8 (0.0; 104.2)	4.4	−0.012 (−0.016; −0.008)	<0.001	Whole grain cereals, olives, beer
5-Pentacosylresorcinol	1.0 (0.0; 5.8)	8.7	−0.011 (−0.017; −0.005)	0.002	Olives, legumes, bread
5-Tricosylresorcinol	2.4 (0.2; 8.8)	0.8	−0.034 (−0.044; −0.025)	<0.001	Coffee, beer, cream desserts
Apigenin 6,8-C-arabinoside-C-glucoside	1.8 (0.3; 7.6)	1.0	−0.023 (−0.032; −0.012)	<0.001	Wine, apples, pears
Apigenin 6,8-C-galactoside-C-arabinoside	2.5 (0.4; 9.6)	1.0	−0.023 (−0.033; −0.014)	<0.001	Vegetable oils, olives, sauces
Apigenin 6,8-di-C-glucoside	2.1 (0.1; 10.0)	2.1	−0.017 (−0.023; −0.011)	<0.001	FV, olives
Caffeic acid	1.9 (0.7; 6.5)	0.0	−0.058 (−0.084; −0.033)	<0.001	Coffee, cream desserts, beer
Caffeoyl tartaric acid	1.1 (0.0; 9.4)	0.7	−0.021 (−0.028; −0.015)	<0.001	LV, berries, FV
Cyanidin 3-O-glucoside	1.4 (0.1; 9.4)	0.2	−0.021 (−0.030; −0.009)	<0.001	Olives, berries
Cyanidin 3-O-rutinoside	4.0 (0.0; 48.9)	2.4	−0.007 (−0.013; −0.002)	0.009	Olives, sauces, mixed vegetables
Delphinidin 3-O-glucoside	0.7 (0.0; 5.4)	2.1	−0.007 (−0.015; −0.002)	0.024	Soya products, vegetarian dishes
Delphinidin 3-O-rutinoside	0.2 (0.0; 13.8)	17.6	−0.007 (−0.011; −0.003)	<0.001	Whole grain cereals, rice
Didymin	2.0 (0.1 9.2)	2.1	−0.017 (−0.023; −0.011)	<0.001	Wine, olives, beer
Dihydromyricetin 3-O-rhamnoside	0.5 (0.0; 10.4)	10.0	−0.013 (−0.017; −0.010)	<0.001	Wine, apples, pears
Ellagic acid	0.9 (0.0; 11.5)	1.9	−0.024 (−0.031; −0.018)	<0.001	Wine, cocktails, sauces
Ferulic acid	22.5 (0.2; 134.0)	0.0	−0.033 (−0.051; −0.014)	<0.001	Whole grain cereals, olives
Gallic acid	5.6 (0.2; 43.9)	0.1	−0.016 (−0.024; −0.009)	<0.001	Vegetable oils, sauces, bread
Hesperidin	16.5 (0.9; 9.2)	0.8	−0.017 (−0.023; −0.011)	<0.001	Citrus fruits, beer, olives
Kaempferol 3-O-glucoside	1.8 (0.1; 10.5)	0.3	−0.024 (−0.034; −0.015)	<0.001	Mixed vegetables, soups
Malvidin 3-O-(6-p-coumaroyl-glucoside)	0.7 (0.0; 13.5)	6.4	−0.008 (−0.012; −0.003)	<0.001	Berries, cakes, pastries
Naringin	0.5 (0.0; 9.0)	0.8	−0.020 (−0.026; −0.014)	<0.001	Legumes, soups, citrus fruits
Narirutin	2.8 (0.1; 14.2)	0.9	−0.017 (−0.024; −0.012)	<0.001	FJ, citrus fruits, soft drinks
O-Coumaric acid	0.7 (0.0; 5.2)	2.8	−0.004 (−0.009; 0.002)	0.178	Mixed vegetables, berries, LV
Oleuropein-aglycone	0.2 (0.0; 4.9)	1.0	−0.016 (−0.022; −0.009)	<0.001	Wine, sauces
P-Courmaric acid	2.2 (0.5; 6.1)	0.0	−0.003 (−0.022; 0.015)	0.733	Soups, berries, legumes
Pelargonidin 3-O-glucoside	2.2 (0.1; 13.5)	1.4	−0.012 (−0.019; −0.004)	0.003	FV, legumes
Phloridzin	1.0 (0.1; 3.8)	1.7	−0.024 (−0.032; −0.018)	<0.001	Berries, soft drinks, FJ
Phlorin	0.6 (0.0; 3.3)	1.6	−0.016 (−0.022; −0.009)	<0.001	LV, whole grain cereals, rice
Proanthocyanidin Polymers (>10 mers)	69.0 (18.2; 198.5)	0.1	−0.000 (−0.016; 0.014)	0.952	Spices, herbs, soups
Proanthocyanidins 04–06 oligomers	42.0 (11.0; 119.2)	0.1	−0.005 (−0.020; 0.011)	0.608	Sweets, bread, seeds
Proanthocyanidins 07–10 oligomers	30.0 (7.6; 82.8)	0.1	−0.011 (−0.026; 0.004)	0.178	Sweets, bread, seeds
Procyanidin dimer B3	3.4 (0.3; 23.2)	0.0	−0.056 (−0.068; −0.045)	<0.001	Berries, jams
Procyanidin dimer B4	3.9 (0.0; 23.9)	0.5	−0.030 (−0.036; −0.023)	<0.001	LV, vegetarian products
Procyanidin dimer B7	1.9 (0.2; 7.2)	0.4	−0.046 (−0.056; −0.035)	<0.001	Wine, spirits, cocktails
Prodelphinidin dimer B3	0.6 (0.0;14.9)	0.5	−0.019 (−0.025; −0.012)	<0.001	Sweets, bread, seeds
Protocatechuic acid	0.5 (0.1; 4.7)	0.0	0.006 (−0.010; 0.022)	0.434	FV, mixed vegetables, soups
Quercetin	1.2 (0.2; 4.9)	0.0	−0.041 (−0.056; −0.028)	<0.001	Beer, peanuts, mixed fruits
Quercetin 3,4-O-diglucoside	1.7 (0.3; 8.7)	0.2	−0.011 (−0.023; 0.002)	0.100	Spices, herbs, sauces
Quercetin 3-O-galactoside	1.5 (0.1; 7.9)	0.1	−0.049 (−0.058; −0.039)	<0.001	Berries, sweets, bread
Quercetin 3-O-glucoside	3.7 (0.3; 13.0)	0.0	−0.062 (−0.074; −0.051)	<0.001	FV, legumes
Quercetin 3-O-rhamnoside	1.1 (0.2; 3.9)	0.0	−0.071 (−0.085; −0.056)	<0.001	Beer, cocktails
Quercetin 3-O-rutinoside	0.4 (0.4; 16.6)	0.0	−0.050 (−0.064; −0.038)	<0.001	Soup, bread, flours
Quercetin 4-O-glucoside	1.2 (0.2; 6.3)	0.2	−0.011 (−0.023; 0.001)	0.091	Berries, jams
Sanguiin H-6	0.1 (0.0; 4.2)	48.6	0.000 (−0.005; 0.007)	0.523	Sauces, condiments, soups
Sinapic acid	0.73 (0.11; 4.15)	0.1	0.021 (0.009; 0.033)	<0.001	Olives, FV, legumes
Stigmastanol ferulate	0.7 (0.0; 9.6)	16.1	0.004 (−0.000; 0.008)	0.074	Wine, spirits, cocktails
Tyrosol	1.1 (0.0; 8.5)	0.1	−0.038 (−0.046; −0.029)	<0.001	Mixed fruits

P5 and p95: percentile 5th and 95th; FJ: fruit juices; FV: fruiting vegetables; LV: leafy vegetables; N-C: non-consumers; SF: stone fruits. Overall mean 5-year weight gain corresponded to 2.6 (5.0) kg and negative beta-values indicate less weight gain (kg) over 5 years based on log2-transformed polyphenol intakes. ^1^ Selection criteria: consumers mean ≥ 1 mg/d; consumers ≥ 50%; Pearson’s correlation coefficient < 0.8. ^2^ Multilevel linear mixed models with random effects on the intercept according to EPIC centre adjusted for age, sex, body mass index (3-knot restricted cubic spline), follow-up time in years (3-knot restricted cubic spline), alcohol intake at baseline (g/d), education level, physical activity level, smoking status at follow-up, menopausal status, total energy intake (kcal/d), plausibility of energy intake reporting, vitamin C intake (mg/d), and fibre intake (g/d). ^3^ false discovery rate *q* value considered statistically significant at ≤0.05. ^4^ Main food sources in descending order of polyphenol content according to our previous EPIC study.

## Data Availability

For information on how to submit an application for gaining access to EPIC data and/or biospecimens, please follow the instructions at http://epic.iarc.fr/access/index.php (accessed on 1 December 2022).

## Supplementary Materials

The following supporting information can be downloaded at: https://www.mdpi.com/article/10.3390/antiox11122425/s1, Figure S1: Flow chart of the study population; Figure S2: Flowchart of polyphenols selected and key compounds; Table S1: Correlated polyphenols according to Pearson Correlation Coefficient; Table S2: Change in 5-year body weight according to polyphenol intake in 349,165 participants from the EPIC-PANACEA cohort: comparison between models; Table S3: Associations between selected individual polyphenols and body weight change according to coffee consumption in the EPIC-PANACEA cohort; Table S4: Change in 5-year body weight according to polyphenol intake in participants without chronic diseases at baseline or with measured body weight at follow-up; Table S5: Change in 5-year body weight estimating polyphenols according to energy density in 349,165 participants; Table S6: Interactions by sex: change in 5-year body weight according to polyphenol intake in 349,165 participants; Table S7: Interactions by age: change in 5-year body weight according to polyphenol intake in 349,165 participants; Table S8: Interactions by BMI categories: change in 5-year body weight according to polyphenol intake in 349,165 participants; Table S9: Interactions by menopausal status: change in 5-year body weight according to polyphenol intake in 255,730 female participants; Table S10: Interactions by smoking status at follow-up: change in 5-year body weight according to polyphenol intake in 334,616 participants; Table S11: Interactions by tertiles of fibre consumption: change in 5-year body weight according to polyphenol intake in 349,165 participants; Table S12: Individual polyphenols represented in Figure 2.
