# Supplementary material for: Dietary Intake of 91 Individual Polyphenols and 5-Year Body Weight Change in the EPIC-PANACEA Cohort
Source: Antioxidants (Basel). 2022 Dec 8;11(12):2425. doi: 10.3390/antiox11122425 (PMC9774775; doi:10.3390/antiox11122425)
Supplement: Supplementary file 1 [file antioxidants-11-02425-s001.zip › antioxidants-2068249-supplementary.pdf]

Figure S1. Flow chart of the study population

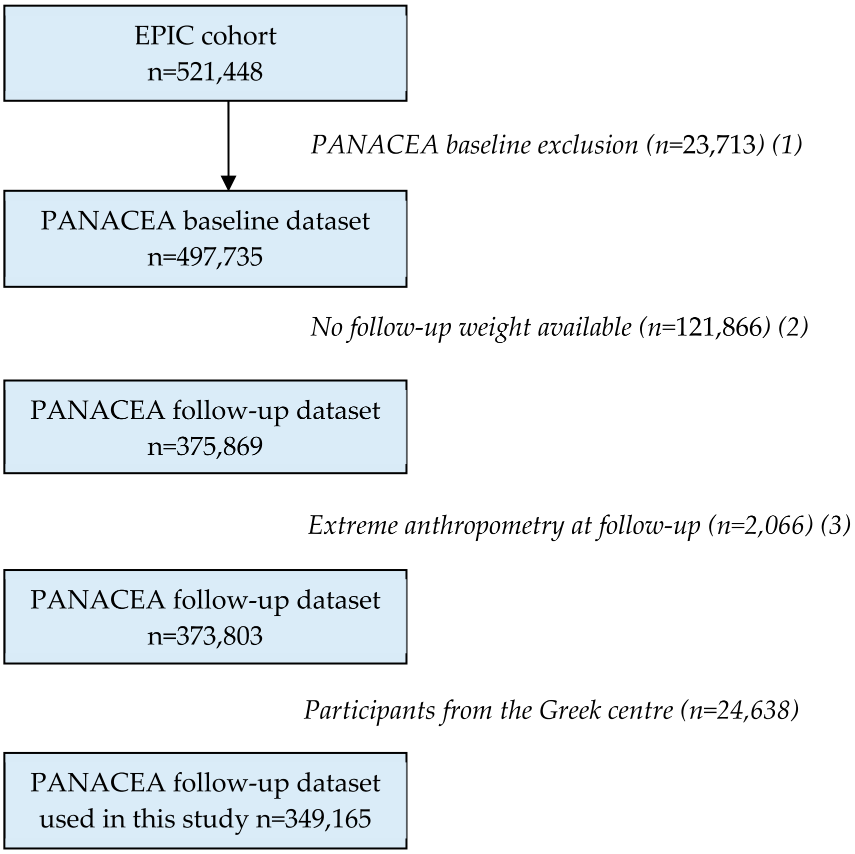

(1) PANACEA baseline exclusions:
   1. Length of follow-up equals to 0 (n=1,517)
   2. No dietary data available (n=6,611)
   3. Extreme ratio of reported energy intake/energy requirement (EI/ER) (n=10,209)
   4. No lifestyle information (n=64)
   5. Pregnancy (n=623)
   6. Unreliable anthropometry (height<1.3 m (n=16), BMI<16.0 kg/m^2 (n=302), waist circumference<40 cm (n=0) or waist circumference>160 cm (n=16), waist circumference<60 cm & BMI>25 kg/m^2 (n=42)).
   7. Missing information on weight (n=4,079)
   8. Missing information on height (n=234)

(2) Reasons for missing data on follow-up assessment of body weight:
   1. Death before the follow-up body weight assessment (n=8,226)
   2. Not yet approached for follow-up body weight assessment (n=23,957)
   3. (E)migrated (n=3,991)
   4. Non-respondents to the invitation to participate in the second follow-up assessment of body weight (n=85,967)
   5. Follow-up time missing (n=13)

(3) Extreme anthropometry at follow-up:
   1. Annual weight change < -5 kg or > 5 kg (n=1,926)
   2. BMI at follow-up < 16 kg/m^2 (n=140)
   3. Missing BMI at follow-up (n=222)

Figure S2. Flowchart of polyphenols selected and key compounds**.**

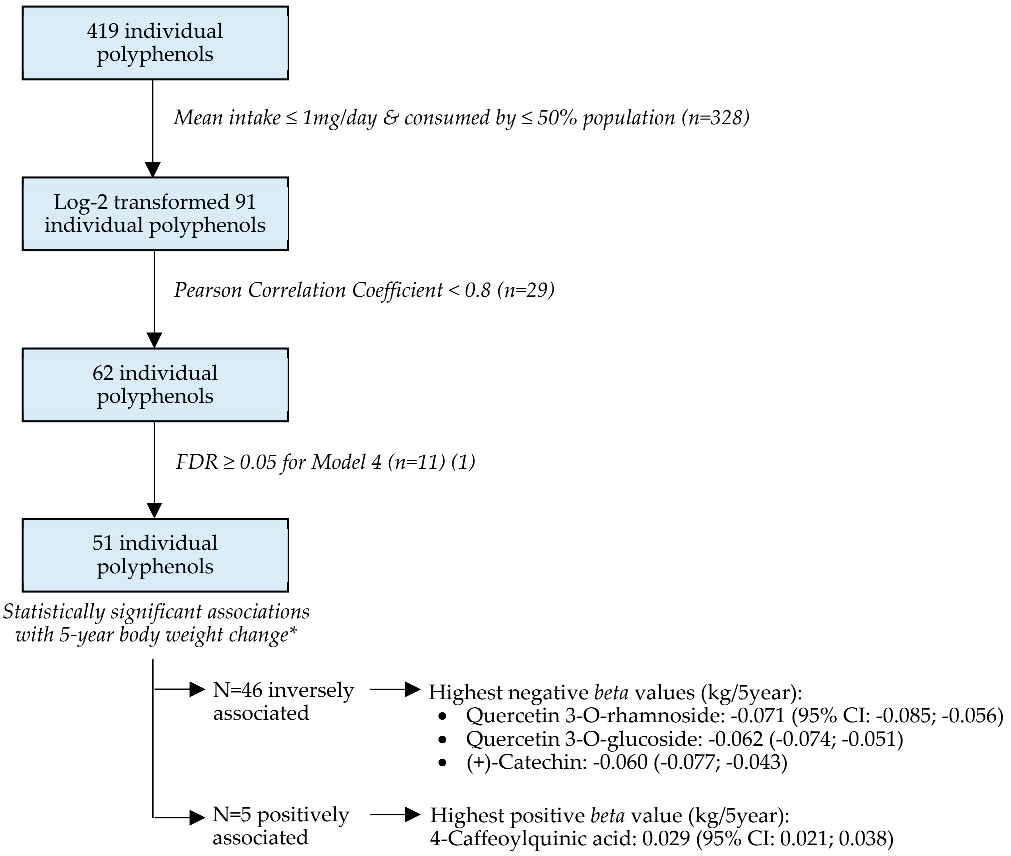

*For doubling in intake of individual polyphenols (log2-transformed)
(1) Multilevel linear mixed model with random effects on the intercept according to EPIC centre adjusted for age, sex, body mass index (3-knot restricted cubic spline), follow-up time in years (3-knot restricted cubic spline), alcohol intake (g/day), education level, physical activity level, smoking status at follow-up, menopausal status, total energy intake (kcal/d), plausibility of dietary energy reporting, vitamin C intake (mg/d), and fibre intake (g/d)

Table S1. Highly correlated polyphenols and their main food sources in the EPIC-PANACEA cohort

Groups of correlated polyphenols (n=35)[1]	Intake (mg/d), median (p5; p95)	N-C (%)	Selected polyphenols (n=6)[2]	Food sources[3]
Malvidin 3-O-(6-acetyl -glucoside)	1.1 (0.0; 7.9)	6.0	Malvidin 3-O-(6-p-coumaroyl-glucoside)	Berries, cakes, pastries
Malvidin 3-O-glucoside	0.8 (0.0; 4.8)	1.8		
Malvidin 3-O-(6-p-coumaroyl-glucoside)	3.9 (0.0; 23.6)	6.4		
Phloretin 2-O-xylosyl-glucoside	0.7 (0.0; 2.9)	1.7	Phloridzin	Berries, soft drinks, fruit juices
Phloridzin	1.0 (0.0; 3.8)	1.7		
Procyanidin trimer T2	0.5 (0.0; 10.3)	23.4	Dihydromyricetin 3-O-rhamnoside	Wine, apples, pears
Dihydromyricetin 3-O-rhamnoside	0.7 (0.0; 15.1)	10.0		
(-)-Epigallocatechin	1.25 (0.0; 64.7)	0.3	(-)-Epicatechin 3-O-gallate	Coffee, tea, stone fruits
(-)-Epigallocatechin 3-O-gallate	1.5 (0.0; 82.0)	3.8		
(-)-Epicatechin 3-O-gallate	3.3 (0.1; 66.2)	0.2		
(+)-Catechin 3-O-gallate	0.8 (0.0; 42.6)	10.2		
(+)-Gallocatechin	1.9 (0.0; 126.1)	0.3		
(+)-Gallocatechin 3-O-gallate	0.2 (0.0; 6.0)	10.1		
(+)-Catechin	10.8 (2.2; 35.7)	0.0	(+)-Catechin	Apples, pears, chocolate
Theaflavin	0.4 (0.0; 29.4)	40.4		
Theaflavin 3,3-O-digallate	0.5 (0.0; 31.7)	40.4		
Theaflavin 3'-O-gallate	0.5 (0.0; 36.7)	40.4		
Theaflavin 3-O-gallate	0.2 (0.0; 14.2)	40.4		
Kaempferol 3-O-glucosyl-rhamnosyl-glucoside	0.1 (0.0; 6.0)	40.4		
Kaempferol 3-O-rutinoside	0.2 (0.0; 11.9)	1.6		
Quercetin 3-O-glucosyl-rhamnosyl-galactoside	0.1 (0.0; 6.0)	40.4		
Quercetin 3-O-glucosyl-rhamnosyl-glucoside	0.1 (0.0; 9.9)	40.4		
(-)-Epicatechin	10.1 (3.3; 52.8)	0.0		

Procyanidin dimer B1	9.7 (1.1; 43.0)	0.0		
Procyanidin dimer B2	10.6 (2.5; 42.9)	0.0		
Procyanidin trimer C1	5.6 (0.8; 16.2)	0.2		
3,4-Dicaffeoylquinic acid	5.2 (0.3; 14.9)	0.2		
3,5-Dicaffeoylquinic acid	3.2 (0.3; 9.2)	0.2		
3-Caffeoylquinic acid	104.4 (7.2; 308.1)	0.0		
4-Caffeoylquinic acid	113.2 (2.9; 328.4)	0.0		
4,5-Dicaffeoylquinic acid	3.8 (0.0; 11.0)	4.2	4-Caffeoylquinic acid	Coffee, tea, legumes
5-Caffeoylquinic acid	107.8 (36.6; 438.5)	0.0		
4-Ethylguaiacol	1.2 (0.0; 3.4)	4.2		
4-Vinylguaiacol	0.9 (0.0; 2.9)	2.2		
Pyrogallol	1.0 (0.0; 2.9)	2.7		

[1]Pearson Correlation Coefficient ≥0.8

[2]Selection criteria: highest median intake.

[3]Main food sources in descending order of polyphenol content according to our previous EPIC study.

Table S2. Change in 5-year body weight according to polyphenol intake in 349,165 participants from the EPIC-PANACEA cohort: comparison between models.

Individual polyphenol (n=62)[a]	Model 1 beta (95% CI)[1]	Model 2 beta (95% CI)[2]	Model 3 beta (95% CI)[3]	Model 4 beta (95% CI)[4]
(-)-Epicatechin 3-O-gallate	-0.022 (-0.027; -0.016)	-0.021 (-0.026; -0.015)	-0.018 (-0.024; -0.013)	-0.018 (-0.023; -0.013)
(+)-Catechin	-0.066 (-0.081; -0.050)	-0.065 (-0.080; -0.048)	-0.060 (-0.077; -0.043)	-0.060 (-0.076; -0.043)
2,5-di-S-Glutathionyl caftaric acid	-0.013 (-0.016; -0.010)	-0.013 (-0.016; -0.010)	-0.012 (-0.016; -0.009)	-0.012 (-0.015; -0.009)
3,4-DHPEA-EDA	-0.026 (-0.033; -0.019)	-0.024 (-0.031; -0.016)	-0.023 (-0.031; -0.016)	-0.022 (-0.031; -0.016)
3-Feruloylquinic acid	0.020 (0.012; 0.028)	0.022 (0.012; 0.032)	0.025 (0.017; 0.033)	0.026 (0.018; 0.033)
3-p-Coumaroylquinic acid	-0.030 (-0.039; -0.020)	-0.030 (-0.039; -0.020)	-0.030 (-0.040; -0.021)	-0.030 (-0.040; -0.021)
4-Caffeoylquinic acid	0.023 (0.015; 0.032)	0.025 (0.015; 0.035)	0.030 (0.021; 0.038)	0.029 (0.021; 0.038)
4-Feruloylquinic acid	0.013 (0.007; 0.019)	0.015 (0.009; 0.021)	0.017 (0.011; 0.023)	0.017 (0.011; 0.023)
4-Hydroxybenzoic acid	-0.031 (-0.039; -0.023)	-0.031 (-0.039; -0.023)	-0.033 (-0.044; -0.023)	-0.033 (-0.044; -0.023)
4-p-Coumaroylquinic acid	-0.019 (-0.025; -0.013)	-0.020 (-0.026; -0.014)	-0.022 (-0.028; -0.016)	-0.022 (-0.027; -0.015)
5-Feruloylquinic acid	0.015 (0.008; 0.021)	0.016 (0.009; 0.023)	0.019 (0.012; 0.025)	0.019 (0.012; 0.025)
5-Heneicosenylresorcinol	-0.000 (-0.004; 0.004)	-0.000 (-0.004; 0.004)	-0.000 (-0.005; 0.004)	-0.000 (-0.005; 0.004)
5-Heneicosylresorcinol	-0.036 (-0.047; -0.026)	-0.037 (-0.049; -0.027)	-0.039 (-0.050; -0.028)	-0.039 (-0.050; -0.028)
5-Heptadecylresorcinol	-0.035 (-0.044; -0.025)	-0.036 (-0.045; -0.027)	-0.038 (-0.048; -0.028)	-0.038 (-0.048; -0.029)
5-Nonadecylresorcinol	-0.035 (-0.045; -0.025)	-0.037 (-0.047; -0.026)	-0.037 (-0.048; -0.027)	-0.037 (-0.048; -0.027)
5-O-Galloylquinic acid	-0.013 (-0.017; -0.010)	-0.013 (-0.017; -0.010)	-0.012 (-0.016; -0.008)	-0.012 (-0.016; -0.008)
5-Pentacosylresorcinol	-0.010 (-0.016; -0.004)	-0.010 (-0.017; -0.005)	-0.011 (-0.018; -0.005)	-0.011 (-0.017; -0.005)
5-Tricosylresorcinol	-0.031 (-0.041; -0.022)	-0.033 (-0.043; -0.024)	-0.034 (-0.044; -0.025)	-0.034 (-0.044; -0.025)
Apigenin 6,8-C-arabinoside-C-glucoside	-0.019 (-0.028; -0.011)	-0.020 (-0.029; -0.012)	-0.023 (-0.032; -0.013)	-0.023 (-0.032; -0.012)
Apigenin 6,8-C-galactoside-C-arabinoside	-0.020 (-0.029; -0.011)	-0.021 (-0.029; -0.011)	-0.023 (-0.033; -0.014)	-0.023 (-0.033; -0.014)
Apigenin 6,8-di-C-glucoside	-0.016 (-0.022; -0.011)	-0.016 (-0.022; -0.011)	-0.017 (-0.023; -0.011)	-0.017 (-0.023; -0.011)
Caffeic acid	-0.059 (-0.078; -0.040)	-0.059 (-0.080; -0.038)	-0.058 (-0.084; -0.033)	-0.058 (-0.084; -0.033)
Caffeoyl tartaric acid	-0.025 (-0.030; -0.019)	-0.023 (-0.028; -0.015)	-0.021 (-0.028; -0.015)	-0.021 (-0.028; -0.015)
Cyanidin 3-O-glucoside	-0.023 (-0.032; -0.013)	-0.022 (-0.032; -0.013)	-0.020 (-0.031; -0.010)	-0.021 (-0.030; -0.009)
Cyanidin 3-O-rutinoside	-0.007 (-0.012; -0.002)	-0.007 (-0.012; -0.002)	-0.007 (-0.012; -0.002)	-0.007 (-0.013; -0.002)

Delphinidin 3-O-glucoside	-0.012 (-0.018; -0.006)	-0.011 (-0.017; -0.006)	-0.008 (-0.015; -0.002)	-0.007 (-0.015; -0.002)
Delphinidin 3-O-rutinoside	-0.007 (-0.011; -0.003)	-0.007 (-0.011; -0.003)	-0.007 (-0.011; -0.003)	-0.007 (-0.011; -0.003)
Didymin	-0.017 (-0.022; -0.011)	-0.017 (-0.022; -0.011)	-0.017 (-0.023; -0.011)	-0.017 (-0.023; -0.011)
Dihydromyricetin 3-O-rhamnoside	-0.014 (-0.017; -0.011)	-0.014 (-0.017; -0.011)	-0.013 (-0.017; -0.010)	-0.013 (-0.017; -0.010)
Ellagic acid	-0.023 (-0.029; -0.017)	-0.023 (-0.029; -0.017)	-0.024 (-0.031; -0.018)	-0.024 (-0.031; -0.018)
Ferulic acid	-0.026 (-0.043; -0.008)	-0.028 (-0.047; -0.011)	-0.033 (-0.051; -0.014)	-0.033 (-0.051; -0.014)
Gallic acid	-0.025 (-0.032; -0.017)	-0.023 (-0.030; -0.015)	-0.017 (-0.025; -0.009)	-0.016 (-0.024; -0.009)
Hesperidin	-0.018 (-0.024; -0.012)	-0.018 (-0.024; -0.012)	-0.017 (-0.023; -0.011)	-0.017 (-0.023; -0.011)
Kaempferol 3-O-glucoside	-0.030 (-0.038; -0.021)	-0.029 (-0.037; -0.020)	-0.024 (-0.034; -0.015)	-0.024 (-0.034; -0.015)
Malvidin 3-O-(6-p-coumaroyl-glucoside)	-0.011 (-0.016; -0.007)	-0.011 (-0.015; -0.007)	-0.008 (-0.013; -0.003)	-0.008 (-0.012; -0.003)
Naringin	-0.023 (-0.029; -0.018)	-0.022 (-0.028; -0.018)	-0.020 (-0.026; -0.014)	-0.020 (-0.026; -0.014)
Narirutin	-0.018 (-0.024; -0.013)	-0.018 (-0.024; -0.013)	-0.018 (-0.024; -0.012)	-0.017 (-0.024; -0.012)
O-Coumaric acid	-0.005 (-0.008; 0.003)	-0.005 (-0.008; 0.003)	-0.004 (-0.009; 0.002)	-0.004 (-0.009; 0.002)
Oleuropein-aglycone	-0.019 (-0.026; -0.013)	-0.017 (-0.025; -0.012)	-0.016 (-0.023; -0.009)	-0.016 (-0.022; -0.009)
P-Coumaric acid	-0.004 (-0.023; 0.014)	-0.004 (-0.023; 0.014)	-0.003 (-0.023; 0.015)	-0.003 (-0.022; 0.015)
Pelargonidin 3-O-glucoside	-0.011 (-0.018; -0.004)	-0.011 (-0.018; -0.004)	-0.012 (-0.019; -0.004)	-0.012 (-0.019; -0.004)
Phloridzin	-0.018 (-0.025; -0.011)	-0.018 (-0.025; -0.011)	-0.025 (-0.032; -0.018)	-0.024 (-0.032; -0.018)
Phlorin	-0.016 (-0.022; -0.010)	-0.016 (-0.022; -0.010)	-0.016 (-0.022; -0.009)	-0.016 (-0.022; -0.009)
Proanthocyanidin Polymers (>10 mers)	-0.000 (-0.015; 0.014)	-0.000 (-0.015; 0.014)	-0.000 (-0.016; 0.014)	-0.000 (-0.016; 0.014)
Proanthocyanidins 04-06 oligomers	-0.004 (-0.019; 0.011)	-0.004 (-0.019; 0.011)	-0.005 (-0.020; 0.011)	-0.005 (-0.020; 0.011)
Proanthocyanidins 07-10 oligomers	-0.011 (-0.026; 0.004)	-0.011 (-0.026; 0.004)	-0.011 (-0.026; 0.004)	-0.011 (-0.026; 0.004)
Procyanidin dimer B3	-0.053 (-0.062; -0.044)	-0.054 (-0.062; -0.044)	-0.057 (-0.069; -0.046)	-0.056 (-0.068; -0.045)
Procyanidin dimer B4	-0.033 (-0.038; -0.027)	-0.033 (-0.037; -0.027)	-0.030 (-0.036; -0.023)	-0.030 (-0.036; -0.023)
Procyanidin dimer B7	-0.043 (-0.053; -0.034)	-0.044 (-0.054; -0.034)	-0.046 (-0.056; -0.035)	-0.046 (-0.056; -0.035)
Prodelphinidin dimer B3	-0.023 (-0.029; -0.016)	-0.022 (-0.029; -0.016)	-0.019 (-0.026; -0.012)	-0.019 (-0.025; -0.012)
Protocatechuic acid	0.005 (-0.011; 0.021)	0.005 (-0.011; 0.021)	0.006 (-0.010; 0.022)	0.006 (-0.010; 0.022)
Quercetin	-0.047 (-0.060; -0.034)	-0.046 (-0.060; -0.033)	-0.042 (-0.057; -0.028)	-0.041 (-0.056; -0.028)
Quercetin 3,4-O-diglucoside	-0.012 (-0.024; 0.002)	-0.012 (-0.024; 0.002)	-0.011 (-0.023; 0.002)	-0.011 (-0.023; 0.002)

Quercetin 3-O-galactoside	-0.044 (-0.053; -0.035)	-0.045 (-0.055; -0.035)	-0.049 (-0.058; -0.039)	-0.049 (-0.058; -0.039)
Quercetin 3-O-glucoside	-0.061 (-0.074; -0.050)	-0.061 (-0.074; -0.050)	-0.062 (-0.074; -0.051)	-0.062 (-0.074; -0.051)
Quercetin 3-O-rhamnoside	-0.069 (-0.081; -0.056)	-0.069 (-0.082; -0.056)	-0.071 (-0.085; -0.056)	-0.071 (-0.085; -0.056)
Quercetin 3-O-rutinoside	-0.058 (-0.070; -0.045)	-0.056 (-0.069; -0.044)	-0.050 (-0.063; -0.037)	-0.050 (-0.064; -0.038)
Quercetin 4-O-glucoside	-0.010 (-0.022; 0.002)	-0.010 (-0.022; 0.002)	-0.011 (-0.023; 0.001)	-0.011 (-0.023; 0.001)
Sanguiin H-6	0.000 (-0.006; 0.007)	0.000 (-0.006; 0.007)	0.000 (-0.005; 0.007)	0.000 (-0.005; 0.007)
Sinapic acid	0.013 (0.002; 0.024)	0.019 (0.006; 0.027)	0.021 (0.009; 0.033)	0.021 (0.009; 0.033)
Stigmastanol ferulate	0.003 (-0.000; 0.007)	0.003 (-0.000; 0.007)	0.004 (-0.000; 0.008)	0.004 (-0.000; 0.008)
Tyrosol	-0.034 (-0.041; -0.028)	-0.035 (-0.043; -0.029)	-0.038 (-0.046; -0.029)	-0.038 (-0.046; -0.029)

[a]Selection criteria: consumers mean ≥ 1 mg/d; consumers ≥ 50%; Pearson correlation coefficient<0.8

[1]Multilevel linear mixed models with random effects on the intercept according to EPIC centres adjusted for age, sex, and body mass index (3-knot restricted cubic spline).

[2]Further adjusted for follow-up time in years (3-knot restricted cubic spline), alcohol intake (g/d), education level, physical activity level, smoking status at follow-up and menopausal status.

[3]Further adjusted for energy intake (kcal/d) and plausibility of energy intake reporting.

[4]Further adjusted for vitamin C intake (mg/d), and fibre intake (g/d).

Table S3. Associations between selected individual polyphenols and 5-year body weight change according to coffee consumption in the EPIC-PANACEA cohort.

Coffee consumers (n=323,751)		
Polyphenol	Model 4 beta (95% IC)[1]	FDR
3-Feruloylquinic acid	0.047 (0.035; 0.059)	<0.001
4-Caffeoylquinic acid	0.054 (0.042; 0.066)	<0.001
4-Feruloylquinic acid	0.040 (0.030; 0.050)	<0.001
5-Feruloylquinic acid	0.040 (0.030; 0.051)	<0.001
Sinapic acid	0.021 (0.009; 0.033)	<0.001
Coffee non-consumers (n=25,414)		
Polyphenol	Model 4 beta (95% IC)[1]	FDR
3-Feruloylquinic acid	-0.015 (-0.061; 0.029)	0.494
4-Caffeoylquinic acid	-0.042 (-0.092; 0.009)	0.111
4-Feruloylquinic acid	0.021 (-0.006; 0.049)	0.124
5-Feruloylquinic acid	0.018 (-0.024; 0.060)	0.401
Sinapic acid	0.048 (0.015; 0.081)	0.005

Overall mean 5-year weight gain corresponded to 2.6 (5.0) kg and negative beta-values indicate less weight gain (kg) over 5 year based on log2-transformed polyphenol intakes.

[1]Multilevel linear mixed models with random effects on the intercept according to EPIC centre adjusted for age, sex, body mass index (3-knot cubic spline), follow-up time in years (3-knot cubic spline), alcohol intake (g/d), education level, physical activity level, smoking status at follow-up, menopausal status, total energy intake (kcal/d), plausibility of dietary intake reporting, vitamin C intake (mg/d), and fibre intake (g/d).

Table S4. Change in 5-year body weight according to polyphenol intake in participants without chronic diseases at baseline or with measured body weight at follow-up in the EPIC-PANACEA cohort.

Individual polyphenols (n=62)[3]	Participants without chronic diseases at baseline[1] (n=291,548)		Participants with measured body weight at follow-up[2] (n=28,653)	
	Model 4 beta (95% CI)[4]	FDR	Model 4 beta (95% CI)[4]	FDR
(-)-Epicatechin 3-O-gallate	-0.017 (-0.022; -0.011)	<0.001	-0.026 (-0.047; -0.005)	0.041
(+)-Catechin	-0.059 (-0.073; -0.037)	<0.001	-0.099 (-0.165; -0.032)	0.017
2,5-di-S-Glutathionyl caftaric acid	-0.007 (-0.010; -0.005)	<0.001	-0.010 (-0.019; -0.000)	0.059
3,4-DHPEA-EDA	-0.015 (-0.022; -0.007)	<0.001	-0.045 (-0.076; -0.013)	0.020
3-Feruloylquinic acid	0.026 (0.017; 0.036)	<0.001	0.039 (0.012; 0.066)	0.020
3-p-Coumaroylquinic acid	-0.029 (-0.038; -0.019)	<0.001	-0.061 (-0.106; -0.015)	0.023
4-Caffeoylquinic acid	0.031 (0.022; 0.040)	<0.001	0.068 (0.028; 0.102)	0.009
4-Feruloylquinic acid	0.019 (0.013; 0.025)	<0.001	0.018 (-0.002; 0.039)	0.059
4-Hydroxybenzoic acid	-0.033 (-0.044; -0.021)	<0.001	0.003 (-0.033; 0.027)	0.851
4-p-Coumaroylquinic acid	-0.014 (-0.019; -0.009)	<0.001	-0.032 (-0.062; -0.002)	0.049
5-Feruloylquinic acid	0.022 (0.015; 0.029)	<0.001	0.022 (0.000; 0.044)	0.428
5-Heneicosenylresorcinol	-0.000 (-0.007; 0.008)	0.873	0.000 (-0.008; 0.008)	0.851
5-Heneicosylresorcinol	-0.039 (-0.050; -0.027)	<0.001	-0.010 (-0.044; 0.024)	0.678
5-Heptadecylresorcinol	-0.026 (-0.034; -0.018)	<0.001	-0.007 (-0.040; 0.026)	0.774
5-Nonadecylresorcinol	-0.036 (-0.047; -0.026)	<0.001	-0.009 (-0.043; 0.025)	0.708
5-O-Galloylquinic acid	-0.011 (-0.014; -0.007)	<0.001	-0.011 (-0.024; -0.000)	0.059
5-Pentacosylresorcinol	-0.006 (-0.011; -0.000)	0.026	-0.003 (-0.027; 0.020)	0.837
5-Tricosylresorcinol	-0.025 (-0.034; -0.017)	<0.001	-0.008 (-0.040; 0.025)	0.725
Apigenin 6,8-C-arabinoside-C-glucoside	-0.019 (-0.028; -0.009)	<0.001	-0.079 (-0.130; -0.028)	0.010
Apigenin 6,8-C-galactoside-C-arabinoside	-0.019 (-0.028; -0.010)	<0.001	-0.085 (-0.138; -0.032)	0.010
Apigenin 6,8-di-C-glucoside	-0.013 (-0.018; -0.008)	<0.001	-0.019 (-0.043; 0.005)	0.049
Caffeic acid	-0.060 (-0.087; -0.032)	<0.001	0.048 (-0.052; 0.148)	0.049

Caffeoyl tartaric acid	-0.017 (-0.023; -0.010)	<0.001	-0.005 (-0.034; 0.025)	0.820
Cyanidin 3-O-glucoside	-0.023 (-0.034; -0.012)	<0.001	-0.047 (-0.089; -0.005)	0.046
Cyanidin 3-O-rutinoside	-0.007 (-0.013; -0.002)	0.011	-0.038 (-0.068; -0.007)	0.034
Delphinidin 3-O-glucoside	-0.005 (-0.011; -0.000)	0.013	-0.014 (-0.039; -0.001)	0.049
Delphinidin 3-O-rutinoside	-0.010 (-0.014; -0.006)	<0.001	-0.014 (-0.027; -0.002)	0.046
Didymin	-0.013 (-0.018; -0.008)	<0.001	-0.019 (-0.043; 0.004)	0.059
Dihydromyricetin 3-O-rhamnoside	-0.008 (-0.011; -0.005)	<0.001	-0.006 (-0.017; 0.004)	0.046
Ellagic acid	-0.014 (-0.019; -0.008)	<0.001	-0.026 (-0.057; 0.004)	0.059
Ferulic acid	-0.031 (-0.051; -0.011)	0.003	0.014 (-0.065; 0.036)	0.689
Gallic acid	-0.015 (-0.024; -0.007)	<0.001	-0.041 (-0.076; -0.005)	0.046
Hesperidin	-0.016 (-0.022; -0.010)	<0.001	-0.015 (-0.042; 0.011)	0.059
Kaempferol 3-O-glucoside	-0.020 (-0.029; -0.011)	<0.001	-0.028 (-0.072; 0.015)	0.059
Malvidin 3-O-(6-p-coumaroyl-glucoside)	-0.004 (-0.008; 0.000)	0.071	0.007 (-0.008; 0.024)	0.046
Naringin	-0.016 (-0.021; -0.010)	<0.001	-0.012 (-0.032; 0.007)	0.049
Narirutin	-0.016 (-0.022; -0.010)	<0.001	-0.018 (-0.043; 0.007)	0.059
O-Coumaric acid	-0.002 (-0.007; 0.004)	0.178	-0.000 (-0.007; 0.007)	0.845
Oleuropein-aglycone	-0.010 (-0.016; -0.003)	0.002	-0.060 (-0.092; -0.027)	0.008
P-Courmaric acid	-0.001 (-0.020; 0.013)	0.773	-0.000 (-0.015; 0.014)	0.845
Pelargonidin 3-O-glucoside	-0.010 (-0.016; -0.003)	0.003	-0.004 (-0.040; 0.032)	0.851
Phloridzin	-0.021 (-0.026; -0.014)	<0.001	0.004 (-0.031; 0.039)	0.845
Phlorin	-0.014 (-0.020; -0.008)	<0.001	-0.015 (-0.041; -0.000)	0.059
Proanthocyanidin Polymers (>10 mers)	-0.000 (-0.015; 0.014)	0.952	-0.000 (-0.017; 0.018)	0.952
Proanthocyanidins 04-06 oligomers	-0.007 (-0.022; 0.009)	0.608	-0.002 (-0.030; 0.028)	0.851
Proanthocyanidins 07-10 oligomers	-0.008 (-0.024; 0.002)	0.178	-0.005 (-0.021; 0.009)	0.851
Procyanidin dimer B3	-0.052 (-0.060; -0.040)	<0.001	-0.064 (-0.117; -0.011)	0.046
Procyanidin dimer B4	-0.026 (-0.031; -0.019)	<0.001	-0.039 (-0.066; -0.012)	0.020
Procyanidin dimer B7	-0.041 (-0.051; -0.030)	<0.001	-0.050 (-0.104; 0.005)	0.049
Prodelphinidin dimer B3	-0.015 (-0.022; -0.008)	<0.001	-0.024 (-0.049; 0.001)	0.059

Protocatechuic acid	0.007 (-0.009; 0.020)	0.434	0.010 (-0.005; 0.023)	0.441
Quercetin	0.042 (-0.057; -0.027)	<0.001	-0.068 (-0.129; -0.007)	0.049
Quercetin 3,4-O-diglucoside	-0.010 (-0.021; 0.001)	0.100	-0.009 (-0.020; 0.001)	0.100
Quercetin 3-O-galactoside	-0.048 (-0.058; -0.037)	<0.001	-0.039 (-0.076; -0.002)	0.049
Quercetin 3-O-glucoside	-0.062 (-0.074; -0.051)	<0.001	-0.041 (-0.050; -0.032)	0.010
Quercetin 3-O-rhamnoside	-0.064 (-0.079; -0.047)	<0.001	-0.029 (-0.099; 0.040)	0.049
Quercetin 3-O-rutinoside	-0.047 (-0.061; -0.032)	<0.001	-0.064 (-0.107; -0.020)	0.020
Quercetin 4-O-glucoside	-0.007 (-0.022; 0.002)	0.091	-0.005 (-0.019; 0.004)	0.113
Sanguiin H-6	0.001 (-0.004; 0.008)	0.523	0.002 (-0.003; 0.009)	0.523
Sinapic acid	0.013 (0.002; 0.025)	0.019	0.124 (0.064; 0.184)	0.003
Stigmastanol ferulate	0.005 (-0.000; 0.009)	0.074	0.003 (-0.001; 0.007)	0.113
Tyrosol	-0.036 (-0.045; -0.027)	<0.001	-0.015 (-0.043; 0.011)	0.049

Overall mean 5-year weight gain corresponded to 2.6 (5.0) kg and negative beta-values indicate less weight gain (kg) over 5 year based on log2-transformed polyphenol intakes.

[1]Diabetes, cancer, stroke or myocardial infarction at recruitment were excluded (n=57,617)

[2]Participants from Doetinchen (The Netherlands) and Cambridge (UK) EPIC centres; participants with self-reported body weight at follow-up were excluded (n=320,512)

[3]Selection criteria: consumers mean ≥1mg/d; consumers ≥50%; Pearson Correlation Coefficient <0.8.

[4]Multilevel linear mixed models with random effects on the intercept according to EPIC centre adjusted for age, sex, body mass index (3-knot restricted cubic spline), follow-up time in years (3-knot restricted cubic spline), alcohol intake (g/d), education level, physical activity level, smoking status at follow-up, menopausal status, total energy intake (kcal/d), plausibility of dietary energy reporting, vitamin C intake (mg/d), and fibre intake (g/d).

Table S5. Change in 5-year body weight estimating polyphenols according to energy density in 349,165 participants from the EPIC-PANACEA cohort

Individual polyphenols (n=50)[1]	Model 4 beta (95% CI)[2]	FDR
(-)-Epicatechin 3-O-gallate	-0.013 (-0.019; -0.008)	<0.001
(+)-Catechin	-0.042 (-0.059; -0.025)	<0.001
2,5-di-S-Glutathionyl caftaric acid	-0.008 (-0.012; -0.005)	<0.001
3,4-DHPEA-EDA	-0.018 (-0.025; -0.010)	<0.001
3-Feruloylquinic acid	0.026 (0.018; 0.034)	<0.001
3-p-Coumaroylquinic acid	-0.024 (-0.034; -0.015)	<0.001
4-Caffeoylquinic acid	0.031 (0.022; 0.039)	<0.001
4-Feruloylquinic acid	0.017 (0.011; 0.023)	<0.001
4-Hydroxybenzoic acid	-0.024 (-0.035; -0.014)	<0.001
4-p-Coumaroylquinic acid	-0.016 (-0.021; -0.010)	<0.001
5-Feruloylquinic acid	0.020 (0.013; 0.026)	<0.001
5-Heneicosenylresorcinol	-0.000 (-0.005; 0.004)	0.873
5-Heneicosylresorcinol	-0.033 (-0.044; -0.022)	<0.001
5-Heptadecylresorcinol	-0.034 (-0.044; -0.024)	<0.001
5-Nonadecylresorcinol	-0.032 (-0.042; -0.021)	<0.001
5-O-Galloylquinic acid	-0.010 (-0.014; -0.006)	<0.001
5-Pentacosylresorcinol	-0.009 (-0.015; -0.002)	<0.001
5-Tricosylresorcinol	-0.029 (-0.039; -0.019)	<0.001
Apigenin 6,8-C-arabinoside-C-glucoside	-0.017 (-0.028; -0.010)	<0.001
Apigenin 6,8-C-galactoside-C-arabinoside	-0.019 (-0.028; -0.010)	<0.001
Apigenin 6,8-di-C-glucoside	-0.014 (-0.019; -0.008)	<0.001
Caffeic acid	-0.051 (-0.084; -0.035)	<0.001
Caffeoyl tartaric acid	-0.015 (-0.022; -0.008)	<0.001
Cyanidin 3-O-glucoside	-0.018 (-0.028; -0.008)	<0.001
Cyanidin 3-O-rutinoside	-0.005 (-0.010; -0.000)	0.044
Delphinidin 3-O-glucoside	-0.006 (-0.013; -0.001)	<0.001
Delphinidin 3-O-rutinoside	-0.007 (-0.011; -0.003)	<0.001
Didymin	-0.014 (-0.020; -0.008)	<0.001
Dihydromyricetin 3-O-rhamnoside	-0.010 (-0.013; -0.006)	<0.001
Ellagic acid	-0.020 (-0.026; -0.013)	<0.001
Ferulic acid	-0.020 (-0.039; -0.001)	0.040
Gallic acid	-0.011 (-0.019; -0.002)	0.014
Hesperidin	-0.015 (-0.021; -0.009)	<0.001
Kaempferol 3-O-glucoside	-0.017 (-0.027; -0.008)	<0.001
Malvidin 3-O-(6-p-coumaroyl-glucoside)	-0.004 (-0.009; 0.001)	0.106
Naringin	-0.016 (-0.022; -0.010)	<0.001
Narirutin	-0.016 (-0.022; -0.010)	<0.001
O-Coumaric acid	-0.004 (-0.009; 0.002)	0.178
Oleuropein-aglycone	-0.010 (-0.017; -0.003)	0.004
P-Courmaric acid	-0.003 (-0.021; 0.015)	0.733
Pelargonidin 3-O-glucoside	-0.010 (-0.017; -0.002)	0.012
Phloridzin	-0.017 (-0.024; -0.010)	<0.001
Phlorin	-0.013 (-0.019; -0.006)	<0.001

Proanthocyanidin Polymers (>10 mers)	-0.000 (-0.016; 0.014)	0.952
Proanthocyanidins 04-06 oligomers	-0.005 (-0.020; 0.011)	0.608
Proanthocyanidins 07-10 oligomers	-0.011 (-0.026; 0.005)	0.178
Procyanidin dimer B3	-0.044 (-0.055; -0.032)	<0.001
Procyanidin dimer B4	-0.023 (-0.030; -0.017)	<0.001
Procyanidin dimer B7	-0.034 (-0.044; -0.024)	<0.001
Prodelphinidin dimer B3	-0.014 (-0.021; -0.007)	<0.001
Protocatechuic acid	0.006 (-0.010; 0.022)	0.434
Quercetin	-0.030 (-0.045; -0.016)	<0.001
Quercetin 3,4-O-diglucoside	-0.011 (-0.023; 0.002)	0.100
Quercetin 3-O-galactoside	-0.038 (-0.018; -0.029)	<0.001
Quercetin 3-O-glucoside	-0.062 (-0.074; -0.051)	<0.001
Quercetin 3-O-rhamnoside	-0.052 (-0.066; -0.037)	<0.001
Quercetin 3-O-rutinoside	-0.037 (-0.051; -0.025)	<0.001
Quercetin 4-O-glucoside	-0.011 (-0.023; 0.001)	0.091
Sanguiin H-6	0.000 (-0.005; 0.007)	0.523
Sinapic acid	0.026 (0.015; 0.038)	<0.001
Stigmastanol ferulate	0.004 (-0.000; 0.008)	0.074
Tyrosol	-0.028 (-0.036; -0.019)	<0.001

Overall mean 5-year weight gain corresponded to 2.6 (5.0) kg and negative beta-values indicate less weight gain (kg) over 5 year based on log2-transformed polyphenol intakes.

[1]Selection criteria: consumers mean ≥1mg/d; consumers ≥50%; Pearson Correlation Coefficient <0.8.

[2]Multilevel linear mixed models with random effects on the intercept according to EPIC centre adjusted for age, sex, body mass index (3-knot restricted cubic spline), follow-up time in years (3-knot restricted cubic spline), alcohol intake (g/d), education level, physical activity level, smoking status at follow-up, menopausal status, total energy intake (kcal/d), plausibility of dietary energy reporting, vitamin C intake (mg/d), and fibre intake (g/d).

Table S6. Change in 5-year body weight according to polyphenol intake by sex in 349,165 participants from the EPIC-PANACEA cohort.

Individual polyphenols (n=29)[1]	Sex		
	Male (n=93 435) beta (95% CI)[2]	Female (n=255 730) beta (95% CI)[2]	p-interaction[3]
(-)-Epicatechin 3-O-gallate	-0.019 (-0.029; -0.009)	-0.012 (-0.018; -0.005)	<0.001
3,4-DHPEA-EDA	-0.007 (-0.020; 0.005)	-0.019 (-0.026; -0.011)	<0.001
3-Feruloylquinic acid	0.013 (-0.002; 0.029)	0.034 (0.024; 0.043)	0.003
4-Caffeoylquinic acid	0.020 (0.003; 0.039)	0.037 (0.027; 0.046)	0.006
4-Hydroxybenzoic acid	-0.003 (-0.021; 0.015)	-0.022 (-0.035; -0.009)	<0.001
4-p-Coumaroylquinic acid	-0.015 (-0.023; -0.008)	-0.008 (-0.014; -0.003)	<0.001
5-Heneicosylresorcinol	-0.055 (-0.077; -0.034)	-0.017 (-0.031; -0.005)	<0.001
5-Heptadecylresorcinol	-0.031 (-0.045; -0.018)	-0.017 (-0.026; -0.007)	0.004
5-Nonadecylresorcinol	-0.052 (-0.072; -0.032)	-0.017 (-0.029; -0.005)	<0.001
5-O-Galloylquinic acid	-0.014 (-0.021; -0.008)	-0.006 (-0.010; -0.002)	<0.001
5-Pentacosylresorcinol	-0.014 (-0.024; -0.003)	0.005 (-0.001; 0.011)	<0.001
5-Tricosylresorcinol	-0.033 (-0.047; -0.019)	-0.014 (-0.024; -0.005)	<0.001
Apigenin 6,8-C-arabinoside-C-glucoside	-0.026 (-0.040; -0.011)	-0.008 (-0.018; 0.003)	<0.001
Apigenin 6,8-C-galactoside-C-arabinoside	-0.026 (-0.041; -0.011)	-0.009 (-0.020; 0.002)	<0.001
Apigenin 6,8-di-C-glucoside	-0.010 (-0.019; -0.001)	-0.016 (-0.022; -0.010)	0.002
Caffeic acid	-0.061 (-0.111; -0.018)	-0.021 (-0.056; 0.014)	<0.001
Cyanidin 3-O-glucoside	-0.020 (-0.038; -0.003)	-0.024 (-0.036; -0.011)	0.022
Cyanidin 3-O-rutinoside	-0.009 (-0.017; -0.001)	-0.003 (-0.009; 0.003)	<0.001
Didymin	-0.010 (-0.019; -0.001)	-0.016 (-0.022; -0.010)	0.004
Ellagic acid	-0.019 (-0.027; -0.010)	-0.012 (-0.019; -0.006)	<0.001
Gallic acid	-0.037 (-0.053; -0.021)	-0.011 (-0.021; -0.002)	<0.001
Hesperidin	-0.016 (-0.028; -0.004)	-0.021 (-0.028; -0.014)	<0.001
Narirutin	-0.016 (-0.028; -0.005)	-0.020 (-0.027; -0.013)	<0.001
Oleuropein-aglycone	-0.007 (-0.018; 0.004)	-0.010 (-0.017; -0.003)	<0.001
Pelargonidin 3-O-glucoside	-0.012 (-0.023; -0.002)	-0.006 (-0.013; 0.001)	<0.001
Phloridzin	-0.012 (-0.022; -0.001)	-0.015 (-0.023; -0.007)	<0.001
Prodelphinidin dimer B3	-0.013 (-0.026; 0.001)	-0.012 (-0.019; -0.005)	0.005
Quercetin 3-O-galactoside	-0.049 (-0.067; -0.031)	-0.035 (-0.047; -0.023)	<0.001
Tyrosol	-0.021 (-0.036; -0.005)	-0.024 (-0.034; -0.014)	<0.001

Overall mean 5-year weight gain corresponded to 2.6 (5.0) kg and negative beta-values indicate less weight gain (kg) over 5 year based on log2-transformed polyphenol intakes.

[1]Selection criteria: consumers mean ≥1mg/d; consumers ≥50%; FDR ≤ 0.05; Pearson Correlation Coefficient <0.8.

[2]Multilevel linear mixed models with random effects on the intercept according to EPIC centre adjusted for age, sex, body mass index (3-knot restricted cubic spline), follow-up time in years (3-knot restricted cubic spline), alcohol intake (g/d), education level, physical activity level, smoking status at follow-up, menopausal status, total energy intake (kcal/d), plausibility of dietary energy reporting, vitamin C intake (mg/d), and fibre intake (g/d).

[3]False Discovery Rate-corrected (q value)

Table S7. Change in 5-year body weight according to polyphenol intake by age groups in 349,165 participants from the EPIC-PANACEA cohort.

Individual polyphenols (n=27)[1]	Age		
	<50 years (n=141 572) beta (95% CI)[2]	≥50 years (n=207 593) beta (95% CI)[2]	p-interaction[3]
(-)-Epicatechin 3-O-gallate	-0.014 (-0.023; -0.005)	-0.020 (-0.027; -0.013)	<0.001
(+)-Catechin	-0.067 (-0.094; -0.039)	-0.059 (-0.082; -0.037)	<0.001
3,4-DHPEA-EDA	-0.029 (-0.042; -0.015)	-0.010 (-0.017; -0.002)	<0.001
3-Feruloylquinic acid	0.016 (0.003; 0.028)	0.033 (0.022; 0.044)	0.009
4-p-Coumaroylquinic acid	-0.010 (-0.017; -0.003)	-0.017 (-0.023; -0.011)	<0.001
5-Heneicosylresorcinol	-0.030 (-0.048; -0.013)	-0.025 (-0.039; -0.011)	<0.001
5-Heptadecylresorcinol	-0.022 (-0.034; -0.009)	-0.018 (-0.028; -0.009)	<0.001
5-Nonadecylresorcinol	-0.030 (-0.047; -0.013)	-0.024 (-0.038; -0.011)	<0.001
5-O-Galloylquinic acid	-0.007 (-0.012; -0.001)	-0.012 (-0.017; -0.008)	<0.001
5-Tricosylresorcinol	-0.020 (-0.033; -0.008)	-0.016 (-0.027; -0.006)	<0.001
Apigenin 6,8-C-arabinoside-C-glucoside	-0.008 (-0.021; 0.006)	-0.023 (-0.035; -0.012)	<0.001
Apigenin 6,8-C-galactoside-C-arabinoside	-0.008 (-0.022; 0.006)	-0.026 (-0.037; -0.014)	<0.001
Caffeic acid	-0.084 (-0.127; -0.041)	-0.021 (-0.055; 0.013)	0.002
Cyanidin 3-O-glucoside	-0.030 (-0.047; -0.014)	-0.024 (-0.037; -0.011)	<0.001
Ellagic acid	-0.010 (-0.017; -0.001)	-0.022 (-0.039; -0.015)	<0.001
Ferulic acid	-0.038 (-0.072; -0.006)	0.003 (-0.021; 0.027)	<0.001
Gallic acid	-0.018 (-0.031; -0.006)	-0.012 (-0.023; -0.001)	<0.001
Kaempferol 3-O-glucoside	-0.014 (-0.028; -0.000)	-0.023 (-0.034; -0.012)	<0.001
Naringin	-0.029 (-0.037; -0.020)	-0.008 (-0.015; -0.001)	<0.001
Oleuropein-aglycone	-0.016 (-0.028; -0.003)	-0.005 (-0.011; 0.002)	<0.001
Phloridzin	-0.012 (-0.021; -0.003)	-0.027 (-0.035; -0.019)	<0.001
Procyanidin dimer B4	-0.026 (-0.035; -0.017)	-0.022 (-0.030; -0.014)	<0.001
Procyanidin dimer B7	-0.036 (-0.051; -0.022)	-0.051 (-0.064; -0.038)	<0.001
Prodelphinidin dimer B3	-0.011 (-0.021; -0.000)	-0.015 (-0.024; -0.008)	<0.001
Quercetin 3-O-galactoside	-0.039 (-0.054; -0.023)	-0.058 (-0.071; -0.046)	<0.001
Sinapic acid	0.026 (0.009; 0.044)	0.033 (0.018; 0.047)	0.019
Tyrosol	-0.049 (-0.064; -0.034)	-0.015 (-0.025; -0.006)	<0.001

Overall mean 5-year weight gain corresponded to 2.6 (5.0) kg and negative beta-values indicate less weight gain (kg) over 5 year based on log2-transformed polyphenol intakes.

[1]Selection criteria: consumers mean ≥1mg/d; consumers ≥50%; FDR q≤0.05; Pearson Correlation Coefficient <0.8.

[2]Multilevel linear mixed models with random effects on the intercept according to EPIC centre adjusted for age, sex, body mass index (3-knot restricted cubic spline), follow-up time in years (3-knot restricted cubic spline), alcohol intake (g/d), education level, physical activity level, smoking status at follow-up, menopausal status, total energy intake (kcal/d), plausibility of dietary energy reporting, vitamin C intake (mg/d), and fibre intake (g/d).

[3]False Discovery Rate-corrected (q value)

Table S8. Change in 5-year body weight according to polyphenol intake by BMI categories in 349,165 participants from the EPIC-PANACEA cohort.

Individual polyphenols (n=35)[1]	BMI categories				p-interaction[3]
	Underweight (n=5 259) *Beta* (95% CI)[2]	Normal weight (n=186 181) *Beta* (95% CI)[2]	Overweight (n=116 874) *Beta* (95% CI)[2]	Obesity (n=40 851) *Beta* (95% CI)[2]	
(-)-Epicatechin 3-O-gallate	-0.026 (-0.058; 0.006)	-0.013 (-0.020; -0.007)	-0.016 (-0.026; -0.006)	0.002 (-0.018; 0.021)	<0.001
(+)-Catechin	-0.129 (-0.233; -0.025)	-0.045 (-0.065; -0.023)	-0.066 (-0.097; -0.035)	0.021 (-0.041; 0.084)	<0.001
2,5-di-S-Glutathionyl caftaric acid	0.007 (-0.007; 0.021)	-0.004 (-0.007; -0.002)	-0.006 (-0.010; -0.002)	-0.000 (-0.009; 0.008)	<0.001
3,4-DHPEA-EDA	-0.007 (-0.043; 0.029)	-0.014 (-0.022; -0.007)	-0.027 (-0.035; -0.010)	-0.013 (-0.040; 0.014)	<0.001
3-p-Coumaroylquinic acid	-0.016 (-0.077; 0.045)	-0.038 (-0.050; -0.026)	-0.023 (-0.038; -0.007)	-0.022 (-0.051; 0.007)	<0.001
4-Hydroxybenzoic acid	0.037 (-0.032; 0.107)	-0.012 (-0.025; 0.001)	-0.037 (-0.056; -0.019)	-0.050 (-0.088; -0.012)	<0.001
4-p-Coumaroylquinic acid	-0.009 (-0.039; 0.021)	-0.012 (-0.017; -0.006)	-0.009 (-0.016; -0.001)	0.003 (-0.013; 0.018)	<0.001
5-O-Galloylquinic acid	-0.015 (-0.033; 0.004)	-0.007 (-0.011; -0.003)	-0.015 (-0.021; -0.009)	-0.000 (-0.013; 0.013)	<0.001
5-Pentacosylresorcinol	-0.009 (-0.044; 0.025)	-0.008 (-0.016; -0.001)	-0.007 (-0.015; 0.002)	0.014 (-0.002; 0.030)	<0.001
Apigenin 6,8-C-arabinoside-C-glucoside	-0.068 (-0.145; 0.009)	-0.033 (-0.047; -0.020)	-0.028 (-0.042; -0.014)	-0.006 (-0.030; 0.017)	<0.001
Apigenin 6,8-C-galactoside-C-arabinoside	-0.072 (-0.151; 0.007)	-0.035 (-0.048; -0.021)	-0.030 (-0.044; -0.015)	-0.007 (-0.030; 0.017)	<0.001
Apigenin 6,8-di-C-glucoside	0.013 (-0.021; 0.047)	-0.016 (-0.023; -0.010)	-0.013 (-0.021; -0.004)	-0.012 (-0.029; 0.004)	<0.001
Caffeic acid	0.170 (0.001; 0.338)	-0.008 (-0.042; 0.025)	-0.080 (-0.127; -0.032)	-0.087 (-181; 0.008)	<0.001
Caffeoyl tartaric acid	-0.035 (-0.086; 0.017)	-0.020 (-0.029; -0.012)	-0.014 (-0.024; -0.004)	0.006 (-0.012; 0.024)	<0.001
Cyanidin 3-O-glucoside	0.029 (-0.010; 0.041)	-0.035 (-0.048; -0.022)	-0.017 (-0.034; 0.001)	-0.004 (-0.035; 0.027)	<0.001
Cyanidin 3-O-rutinoside	0.010 (-0.029; 0.050)	-0.013 (-0.020; -0.006)	-0.009 (-0.017; -0.001)	0.000 (-0.014; 0.017)	<0.001
Delphinidin 3-O-glucoside	0.015 (-0.025; 0.055)	-0.009 (-0.017; -0.002)	-0.002 (-0.011; 0.007)	0.004 (-0.013; 0.021)	<0.001
Delphinidin 3-O-rutinoside	0.009 (-0.011; 0.029)	-0.010 (-0.015; -0.006)	-0.010(-0.016; -0.004)	-0.005 (-0.017; 0.008)	<0.001
Didymin	0.014 (-0.019; 0.048)	-0.017 (-0.023; -0.010)	-0.013 (-0.021; -0.004)	-0.013 (-0.029; 0.004)	<0.001
Dihydromyricetin 3-O-rhamnoside	0.006 (-0.010; 0.022)	-0.004 (-0.007; -0.001)	-0.008 (-0.012; -0.003)	-0.003 (-0.013; 0.006)	<0.001
Ellagic acid	-0.044 (-0.089; 0.002)	-0.029 (-0.037; -0.022)	-0.014 (-0.023; -0.006)	-0.003 (-0.018; 0.013)	<0.001
Ferulic acid	0.045 (-0.062; 0.152)	-0.008 (-0.031; 0.015)	-0.029 (-0.063; 0.006)	-0.072 (-0.147; 0.002)	<0.001

Gallic acid	-0.090 (-0.145; -0.034)	-0.023 (-0.033; -0.012)	-0.020 (-0.034; -0.007)	0.029 (0.004; 0.054)	<0.001
Kaempferol 3-O-glucoside	-0.047 (-0.114; 0.018)	-0.018 (-0.030; -0.007)	-0.024 (-0.038; -0.009)	0.002 (-0.024; 0.028)	<0.001
Malvidin 3-O-(6-p-coumaroyl-glucoside)	-0.006 (-0.031; 0.018)	-0.006 (-0.011; -0.002)	0.000 (-0.006; 0.006)	0.012 (-0.000; 0.024)	<0.001
Naringin	0.012 (-0.018; 0.042)	-0.015 (-0.022; -0.008)	-0.012 (-0.0221; -0.002)	-0.020 (-0.040; -0.000)	<0.001
Pelargonidin 3-O-glucoside	-0.034 (-0.080; 0.012)	-0.014 (-0.022; -0.006)	0.002 (-0.013; 0.008)	-0.013 (-0.031; 0.005)	<0.001
Phloridzin	0.002 (-0.043; 0.047)	-0.024 (-0.032; -0.015)	0.000 (-0.009; 0.010)	-0.010 (-0.029; 0.009)	<0.001
Procyanidin dimer B3	-0.071 (-0.142; 0.001)	-0.043 (-0.057; -0.030)	-0.048 (-0.067; -0.028)	-0.028 (-0.068; 0.013)	<0.001
Procyanidin dimer B4	-0.041 (-0.082; 0.001)	-0.024 (-0.032; -0.016)	-0.023 (-0.033; -0.013)	-0.001 (-0.019; 0.016)	<0.001
Procyanidin dimer B7	-0.037 (-0.102; 0.027)	-0.043 (-0.056; -0.031)	-0.020 (-0.037; -0.003)	-0.017 (-0.049; 0.015)	<0.001
Prodelphinidin dimer B3	-0.012 (-0.047; 0.023)	-0.008 (-0.016; -0.001)	-0.015 (-0.027; -0.004)	-0.008 (-0.030; 0.015)	<0.001
Quercetin 3-O-galactoside	-0.047 (-0.103; 0.010)	-0.038 (-0.050; -0.026)	-0.044 (-0.062; -0.026)	-0.012 (-0.049; 0.026)	<0.001
Quercetin 3-O-rutinoside	-0.080 (-0.155; -0.004)	-0.033 (-0.048; -0.017)	-0.059 (-0.083; -0.034)	-0.006 (-0.059; 0.047)	<0.001
Sinapic acid	0.063 (-0.015; 0.142)	0.043 (0.027; 0.058)	0.011 (-0.009; 0.030)	0.006 (-0.025; 0.037)	<0.001

Overall mean 5-year weight gain corresponded to 2.6 (5.0) kg and negative beta-values indicate less weight gain (kg) over 5 year based on log2-transformed polyphenol intakes.
[1]Selection criteria: consumers mean ≥1mg/d; consumers ≥50%; FDR q≤0.05; Pearson Correlation Coefficient <0.8.
[2]Multilevel linear mixed models with random effects on the intercept according to EPIC centre adjusted for age, sex, body mass index (3-knot restricted cubic spline), follow-up time in years (3-knot restricted cubic spline), alcohol intake (g/d), education level, physical activity level, smoking status at follow-up, menopausal status, total energy intake (kcal/d), plausibility of dietary energy reporting, vitamin C intake (mg/d), fibre intake (g/d) and BMI categories.
[3]False Discovery Rate-corrected (q value)

Table S9. Change in 5-year body weight according to polyphenol intake by menopausal status in 255,730 female participants from the EPIC-PANACEA cohort.

Individual polyphenols (n= 23)[1]	Menopausal status			
	Pre-menopausal (n=83 440) beta (95% CI)[2]	Post-menopausal (n=119 663) beta (95% CI)[2]	Peri-menopausal (n=52 627) beta (95% CI)[2]	p-interaction[3]
(-)-Epicatechin 3-O-gallate	-0.015 (-0.026; -0.003)	-0.016 (-0.025; -0.006)	-0.001 (-0.015; 0.014)	<0.001
(+)-Catechin	-0.050 (-0.086; -0.013)	-0.070 (-0.101; -0.038)	-0.013 (-0.062; 0.035)	<0.001
3,4-DHPEA-EDA	-0.039 (-0.057; -0.020)	-0.018 (-0.027; -0.008)	-0.016 (-0.033; 0.001)	<0.001
3-p-Coumaroylquinic acid	-0.020 (-0.039; -0.000)	-0.043 (-0.060; -0.025)	0.006 (-0.022; 0.035)	0.017
4-p-Coumaroylquinic acid	-0.008 (-0.017; 0.001)	-0.010 (-0.018; -0.002)	-0.009 (-0.022; 0.003)	0.027
5-Heneicosylresorcinol	-0.009 (-0.032; 0.014)	-0.024 (-0.042; -0.006)	-0.011 (-0.042; 0.018)	<0.001
5-Heptadecylresorcinol	-0.008 (-0.025; 0.008)	-0.021 (-0.035; -0.008)	-0.012 (-0.036; 0.012)	0.002
5-Nonadecylresorcinol	-0.009 (-0.031; 0.012)	-0.022 (-0.039; -0.005)	-0.010 (-0.039; 0.018)	<0.001
5-O-Galloylquinic acid	-0.006 (-0.013; 0.000)	-0.009 (-0.014; -0.004)	0.002 (-0.006; 0.011)	<0.001
5-Tricosylresorcinol	-0.007 (-0.024; 0.010)	-0.019 (-0.033; -0.005)	-0.007 (-0.031; 0.017)	<0.001
Delphinidin 3-O-glucoside	0.004 (-0.009; 0.016)	-0.008 (-0.018; -0.001)	-0.010 (-0.028; 0.008)	<0.001
Delphinidin 3-O-rutinoside	-0.006 (-0.038; 0.002)	-0.009 (-0.015; -0.004)	-0.005 (-0.013; 0.003)	<0.001
Dihydromyricetin 3-O-rhamnoside	-0.004 (-0.009; 0.002)	-0.004 (-0.009; 0.000)	-0.008 (-0.015; -0.001)	0.034
Ellagic acid	-0.007 (-0.018; 0.003)	-0.020 (-0.029; -0.010)	-0.000 (-0.018; 0.018)	<0.001
Kaempferol 3-O-glucoside	-0.014 (-0.032; 0.003)	-0.035 (-0.049; -0.019)	-0.006 (-0.033; 0.021)	<0.001
Oleuropein-aglycone	-0.025 (-0.042; -0.008)	-0.010 (-0.019; -0.001)	-0.007 (-0.022; 0.008)	<0.001
Pelargonidin 3-O-glucoside	0.000 (-0.012; 0.012)	-0.012 (-0.023; -0.001)	0.007 (-0.012; 0.026)	0.008
Phloridzin	-0.009 (-0.021; 0.002)	-0.021 (-0.033; -0.009)	-0.023 (-0.043; -0.004)	0.008
Procyanidin dimer B4	-0.020 (-0.032; -0.008)	-0.023 (-0.034; -0.013)	-0.020 (-0.037; -0.002)	<0.001
Procyanidin dimer B7	-0.031 (-0.049; -0.011)	-0.049 (-0.067; -0.032)	-0.025 (-0.054; 0.004)	0.006
Prodelphinidin dimer B3	-0.005 (-0.017; 0.007)	-0.019 (-0.029; -0.009)	-0.005 (-0.021; 0.011)	<0.001
Quercetin 3-O-galactoside	-0.042 (-0.062; -0.021)	-0.043 (-0.060; -0.025)	-0.017 (-0.042; 0.009)	<0.001

Quercetin 3-O-rutinoside	-0.044 (-0.071; -0.017)	-0.053 (-0.078; -0.030)	-0.005 (-0.042; 0.031)	0.001

Overall mean 5-year weight gain corresponded to 2.6 (5.0) kg and negative beta-values indicate less weight gain (kg) over 5 year based on log2-transformed polyphenol intakes.
[1]Selection criteria: consumers mean ≥1mg/d; consumers ≥50%; FDR q ≤ 0.05; Pearson Correlation Coefficient <0.8.
[2]Multilevel linear mixed models with random effects on the intercept according to EPIC centre adjusted for age, sex, body mass index (3-knot restricted cubic spline), follow-up time in years (3-knot restricted cubic spline), alcohol intake (g/d), education level, physical activity level, smoking status at follow-up, menopausal status, total energy intake (kcal/d), plausibility of dietary energy reporting, vitamin C intake (mg/d), and fibre intake (g/d).
[3]False Discovery Rate-corrected (q value)

Table S10. Change in 5-year body weight according to polyphenol intake by smoking status at follow-up in 334,616 participants from the EPIC-PANACEA cohort.

Individual polyphenols (n= 9)[1]	Smoking status at follow-up			p-interaction[3]
	Never (n=168 759) beta (95% CI)[2]	Former (n=105 337) beta (95% CI)[2]	Current (n=60 520) beta (95% CI)[2]	
3-Feruloylquinic acid	0.012 (0.001; 0.023)	0.064 (0.047; 0.074)	-0.011 (-0.033; 0.011)	0.005
4-Caffeoylquinic acid	0.017 (0.006; 0.027)	0.074 (0.056; 0.091)	-0.010 (-0.035; 0.014)	0.008
4-Feruloylquinic acid	0.011 (0.003; 0.017)	0.040 (0.028; 0.052)	-0.013 (-0.029; 0.004)	0.002
4-Hydroxybenzoic acid	-0.018 (-0.034; -0.002)	-0.059 (-0.079; -0.040)	-0.025 (-0.049; -0.002)	0.012
5- Feruloylquinic acid	0.011 (0.003; 0.019)	0.047 (0.034; 0.059)	-0.012 (-0.031; 0.006)	0.004
Pelargonidin 3-O-glucoside	-0.011 (-0.019; -0.002)	-0.021 (-0.033; -0.008)	0.007 (-0.005; 0.019)	0.046
Phloridzin	-0.007 (-0.016; 0.002)	-0.061 (-0.073; -0.050)	0.008 (-0.003; 0.019)	0.002
Procyanidin dimer B7	-0.021 (-0.035; -0.006)	-0.107 (-0.126; -0.088)	0.005 (-0.013; 0.023)	0.002
Quercetin 3-O-galactoside	-0.017 (-0.031; -0.002)	-0.120 (-0.137; -0.102)	-0.004 (-0.023; 0.015)	0.001

Overall mean 5-year weight gain corresponded to 2.6 (5.0) kg and negative beta-values indicate less weight gain (kg) over 5 year based on log2-transformed polyphenol intakes.

[1]Selection criteria: consumers mean ≥1mg/d; consumers ≥50%; FDR q ≤ 0.05; Pearson Correlation Coefficient <0.8.

[2]Multilevel linear mixed models with random effects on the intercept according to EPIC centre adjusted for age, sex, body mass index (3-knot restricted cubic spline), follow-up time in years (3-knot restricted cubic spline), alcohol intake (g/d), education level, physical activity level, smoking status at follow-up, menopausal status, total energy intake (kcal/d), plausibility of dietary energy reporting, vitamin C intake (mg/d), and fibre intake (g/d).

[3]False Discovery Rate-corrected (q value)

Table S11. Change in 5-year body weight according to polyphenol intake by tertiles of fibre consumption in 349,165 participants from the EPIC-PANACEA cohort.

Individual polyphenols (n= 30)[1]	Tertiles of fibre consumption			
	Tertile 1 (≤19g/d) (n=115 225) beta (95% CI)[2]	Tertile 2 (>19 to 25 g/d) (n=115 223) beta (95% CI)[2]	Tertile 3 (>25 g/d) (n=118 717) beta (95% CI)[2]	p-interaction[3]
(-)-Epicatechin 3-O-gallate	-0.019 (-0.028; -0.010)	-0.005 (-0.015; 0.004)	-0.017 (-0.027; -0.008)	<0.001
3,4-DHPEA-EDA	-0.016 (-0.027; -0.005)	-0.026 (-0.038; -0.015)	-0.012 (-0.024; -0.001)	<0.001
3-p-Coumaroylquinic acid	-0.024 (-0.038; -0.010)	-0.020 (-0.037; -0.003)	-0.046 (-0.063; -0.028)	0.034
4-p-Coumaroylquinic acid	-0.012 (-0.019; -0.006)	-0.013 (-0.021; -0.005)	-0.015 (-0.024; -0.005)	0.022
5-Heneicosylresorcinol	-0.025 (-0.044; -0.006)	-0.038 (-0.059; -0.018)	-0.031 (-0.048; -0.013)	<0.001
5-Heptadecylresorcinol	-0.020 (-0.033; -0.007)	-0.033 (-0.047; -0.018)	-0.019 (-0.032; -0.006)	<0.001
5-Nonadecylresorcinol	-0.024 (-0.041; -0.006)	-0.036 (-0.055; -0.017)	-0.030 (-0.046; -0.013)	<0.001
5-O-Galloylquinic acid	-0.011 (-0.017; -0.006)	-0.005 (-0.011; 0.000)	-0.011 (-0.017; -0.006)	<0.001
5-Pentacosylresorcinol	0.003 (-0.007; 0.013)	-0.004 (-0.013; 0.006)	-0.008 (-0.016; -0.000)	<0.001
5-Tricosylresorcinol	-0.019 (-0.032; -0.006)	-0.032 (-0.047; -0.016)	-0.018 (-0.031; -0.004)	<0.001
Apigenin 6,8-C-arabinoside-C-glucoside	-0.020 (-0.036; -0.004)	-0.018 (-0.034; -0.002)	-0.016 (-0.030; -0.002)	0.004
Apigenin 6,8-C-galactoside-C-arabinoside	-0.022 (-0.038; -0.005)	-0.019 (-0.036; -0.004)	-0.017 (-0.031; -0.003)	0.004
Apigenin 6,8-di-C-glucoside	-0.010 (-0.018; -0.002)	-0.016 (-0.026; -0.007)	-0.024 (-0.033; -0.013)	0.005
Caffeic acid	-0.071 (-0.0113; -0.028)	-0.044 (-0.093; 0.003)	0.011 (-0.037; 0.060)	<0.001
Cyanidin 3-O-rutinoside	-0.002 (-0.01; 0.006)	-0.007 (-0.016; 0.001)	-0.013 (-0.022; -0.005)	0.009
Delphinidin 3-O-rutinoside	-0.009 (-0.015; -0.004)	-0.010 (-0.016; -0.004)	-0.007 (-0.013; -0.001)	<0.001
Didymin	-0.010 (-0.018; -0.002)	-0.017 (-0.026; -0.007)	-0.024 (-0.037; -0.014)	0.008
Ellagic acid	-0.010 (-0.019; -0.000)	-0.009 (-0.019; -0.000)	-0.027 (-0.036; -0.018)	<0.001
Ferulic acid	-0.032 (-0.065; -0.000)	-0.037 (-0.071; -0.002)	0.005 (-0.028; 0.037)	0.021
Gallic acid	-0.028 (-0.041; -0.014)	0.003 (-0.011; 0.017)	-0.010 (-0.024; 0.003)	0.013
Hesperidin	-0.014 (-0.024; -0.004)	-0.023 (-0.034; -0.011)	-0.028 (-0.039; -0.016)	0.008
Narirutin	-0.013 (-0.022; -0.003)	-0.024 (-0.035; -0.013)	-0.028 (-0.039; -0.016)	0.007

Oleuropein-aglycone	-0.011 (-0.021; -0.001)	-0.019 (-0.029; -0.009)	-0.006 (-0.016; 0.003)	<0.001
Pelargonidin 3-O-glucoside	-0.007 (-0.017; 0.002)	-0.003 (-0.015; 0.007)	-0.017 (-0.028; -0.006)	<0.001
Phloridzin	-0.012 (-0.022; -0.003)	-0.023 (-0.034; -0.012)	-0.020 (-0.064; -0.008)	0.006
Prodelphinidin dimer B3	-0.019 (-0.029; -0.008)	-0.005 (-0.016; 0.006)	-0.010 (-0.022; 0.001)	0.006
Quercetin 3-O-galactoside	-0.042 (-0.057; -0.027)	-0.042 (-0.059; -0.023)	-0.052 (-0.072; -0.032)	<0.001
Quercetin 3-O-rhamnoside	-0.065 (-0.086; -0.043)	-0.050 (-0.078; -0.022)	-0.045 (-0.076; -0.014)	0.002
Sinapic acid	-0.006 (-0.024; 0.011)	0.037 (-0.017; 0.057)	0.049 (0.028; 0.069)	<0.001
Tyrosol	-0.030 (-0.043; -0.017)	-0.027 (-0.042; -0.013)	-0.029 (-0.045; -0.014)	0.011

Overall mean 5-year weight gain corresponded to 2.6 (5.0) kg and negative beta-values indicate less weight gain (kg) over 5 year based on log2-transformed polyphenol intakes.

[1]Selection criteria: consumers mean ≥1mg/d; consumers ≥50%; FDR q ≤ 0.05; Pearson Correlation Coefficient <0.8.

[2]Multilevel linear mixed models with random effects on the intercept according to EPIC centre adjusted for age, sex, body mass index (3-knot restricted cubic spline), follow-up time in years (3-knot restricted cubic spline), alcohol intake (g/d), education level, physical activity level, smoking status at follow-up, menopausal status, total energy intake (kcal/d), plausibility of dietary energy reporting, vitamin C intake (mg/d), fibre intake (g/d) and tertiles of fibre intake.

[3]False Discovery Rate-corrected (q value)

Table S12. List of individual polyphenols represented in Figure 2.

Reference	Individual polyphenol
1	5-Tricosylresorcinol
2	5-Nonadecylresorcinol
3	5-Henaicosylresorcinol
4	5-Pentacosylresorcinol
5	5-Heptadecylresorcinol
6	Ferulic acid
7	4-Caffeoylquinic acid
8	3,5-Dicaffeoylquinic acid
9	4-Ethylguaiacol
10	4,5-Dicaffeoylquinic acid
11	5-Feruloylquinic acid
12	4-Feruloylquinic acid
13	Pyrogallol
14	3-Feruloylquinic acid
15	3,4-Dicaffeyolquinic acid
16	5-Caffeoylquinic acid
17	3-Caffeoylquinic acid
18	4-Vinylguaiacol
19	Stigmastanol ferulate
20	Ellagic acid
21	Apigenin 6,8-C-galactoside-C-arabinoside
22	Apigenin 6,8-C-arabinoside-C-glucoside
23	Apigenin 6,8-di-C-glucoside
24	Didymin
25	Phlorin
26	Narirutin
27	Hesperidin
28	Sinapic acid
29	o-Coumaric acid
30	Quercetin 4-O-glucoside
31	Quercetin 3,4-O-diglucoside
32	Oleuropein-aglycone
33	3,4 DHPEA-EDA
34	Cyanidin 3-O-rutinoside
35	Cyanidin 3-O-glucoside
36	Pelargonidin 3-O-glucoside
37	Delphinidin 3-O-rutinoside
38	Delphinidin 3-O- glucoside
39	3-p-Coumaroylquinic acid
40	Sanguiin H-6
41	Protocatechuic acid
42	Naringin
43	Quercetin
44	p-Coumaric acid
45	4-Hydroxybenzoic acid
46	Phloridzin
47	Phloretin 2-O-xylosyl-glucoside
48	5-Heneicosenylresorcinol
49	Proanthocyanidin Polymers (>10 mers)
50	Proanthocyanidins 07-10 oligomers
51	Proanthocyanidins 04-06 oligomers
52	2,5 di-S-Glutathionyl caftaric acid
53	Procyanidin trimer T2
54	Dihydromyricetin 3-O-rhamnoside
55	Caffeoyl tartaric acid
56	Tyrosol
57	Malvidin 3-O-glucoside
58	Malvidin 3-O-(6-p-coumaroyl-glucoside)
59	Malvidin 3-O-(6-acetyl-glucoside)
60	Caffeic acid
61	Quercetin 3-O-rhamnoside
62	Procyanidin dímer B3

63	Procyanidin trimer C1
64	Procyanidin dimer B7
65	Procyanidin dimer B1
66	(-)-Epicatechin
67	Procyanidin dimer B2
68	Procyanidin dimer B4
69	(-)-Catechin
70	4-p-Coumaroylquinic acid
71	Quercetin-3-O-galactoside
72	5-O-Galloylquinic acid
73	(+)-Gallocatechin-3-O-gallate
74	Theaflavin-3′-O-gallate
75	Theaflavin
76	Quercetin 3-O-glucosyl-rhamnosyl-glucoside
77	Theaflavin 3,3-O-digallate
78	Theaflavin-3-O-gallate
79	Quercetin 3-O-glucosyl-rhamnosyl-galactoside
80	Kaempferol 3-O-glucosyl-rhamnosyl-glucoside
81	(+)-Catechin-3-O-gallate
82	(+)-Gallocatechin
83	Prodelphinidin dimer B3
84	(-)-Epigallocatechin-3-O-gallate
85	(-)-Epigallocatechin
86	(-)-Epicatechin-3-O-gallate
87	Kaempferol-3-O-rutinoside
88	Quercetin 3-O- rutinoside
89	Kaempferol-3-O-glucoside
90	Gallic acid
91	Quercetin-3-O-glucoside
